# Supplementary material for: Functional Consequences of Splicing of the Antisense Transcript COOLAIR on FLC Transcription
Source: Mol Cell. 2014 Apr 10;54(1):156–65. doi: 10.1016/j.molcel.2014.03.026 (PMC3988885; doi:10.1016/j.molcel.2014.03.026)

Molecular Cell, Volume *54*

## Supplemental Information

# Functional Consequences of Splicing of the Antisense Transcript *COOLAIR* on *FLC* Transcription

Sebastian Marquardt, Oleg Raitskin, Zhe Wu, Fuquan Liu, Qianwen Sun, and Caroline Dean

**Supplemental figures legends**

**Figure S1. The *sof81* mutant maps to the bottom of chromosome 1 and carries a mutation in *PRP8* (*At1g80070)* that does not destabilize protein levels, related to Figure 1.** (A) Mapping of the *sof81* mutation showing number of recombinant chromosomes. "MK41/42" represents an example of a polymorphic marker, at position 29.957 kb from the top of chromosome 1. High-resolution mapping is shown below. The *sof81* mutation maps between MK61/62 and MK55/56. The star (*) at MK55/56 indicates the recombinant chromosome is a different chromosome than that identified with MK61/62. (B) A dCAPS marker verifies the mutation in *sof81*. PCR amplified and BglII digested fragments are shown. The *sof81* G to A transition leads to cleavage of the PCR product. (C) Analysis of PRP8 protein levels by western blotting using the BMR00434 antibody, two biological repeats are shown. Equal loading of total protein is visualized by Coomassie staining of the membrane. (D) Gene expression analysis of the two homologous *Arabidopsis PRP8* copies. *At1g80070/SUS2* expression throughout Arabidopsis developmental stages is shown in green, *At4g38780* expression in red. Note that *At1g80070/SUS2/SOF81* expression is around 100 times higher in most stages. The figure was generated at http://jsp.weigelworld.org/expviz/expviz.jsp (Schmid et al., 2005).

**Figure S2. The *prp8-6* mutation in *sof81* causes the *FLC* expression and flowering time phenotype, related to Figure 1.** (A) The *prp8-6* mutation in PRP8 is required to cause the *FLC* de-repression phenotype. Genomic *PRP8* using endogenous *PRP8* promoter and terminator was cloned and transformed into *prp8-6*. FLC-LUC bioluminescence activity was determined in the transformed plants in the T1 generation. The activity of several transformants (n=12) was quantified and compared to the activity of *prp8-6* mutants (n=5) in the same experiment. A representative image displaying FLC-LUC bioluminescence activity is shown on top, quantification data is shown below as mean +/- SEM. Student's t-test showed P-value $< 0.05$. Warm colours (here red in *prp8-6*) indicate high FLC-LUC bioluminescence activity. High FLC-LUC activity is reduced if a wild-type PRP8 copy is introduced into *prp8-6* through transformation. (B) The *prp8-6* mutation in *PRP8* is sufficient to cause the *FLC* de-repression phenotype. F1 complementation tests based on FLC-LUC bioluminescence activity in *Arabidopsis* seedlings (2 weeks post germination, grey) grown on plates between *prp8-6* and the strong *sus2-4* and *sus2-5 prp8* alleles. Plants heterozygous for the *sus2* T-DNA insertion alleles were crossed to *prp8-6* or C2. The F1 involving *prp8-6* but not C2 segregated for high FLC-LUC bioluminescence activity, demonstrating that the gene disrupted by the *prp8-6* mutation is allelic to gene disrupted by the *sus2* mutations. Genotyping of the individuals (results

indicated on top) confirmed that only the heteroallelic combinations with one copy of the *sus2* allele and one copy of *prp8-6* show elevated FLC-LUC bioluminescence (high activity is shown by meristematic false colouring in warm tones). Note that individuals carrying the L*er* *PRP8* wild-type copy (of the C2 parent) have low FLC-LUC bioluminescence, similar to homozygous C2. The low activity is independent of either allele contributed by the *sus2* parent (either Col *PRP8* wild-type or the *sus2* T-DNA). (C) Complementation test based on flowering time between *prp8-6* and *sus2* alleles. Flowering time analysis of individuals as in (C) assayed by counting the number of rosette leaves before bolting given as means +/- SEM. The allelic combination of *prp8-6* and *sus2* resulted in the most severe late flowering phenotype. Flowering time data for genotypes as annotated below. For the comparisons: *PRP8*-Col/*prp8*-*sof81* vs *prp8*-*sus2-4*/*prp8*-*sof81*; *PRP8*-Col/*prp8*-*sof81* vs *prp8*-*sus2-5*/*prp8*-*sof81*; *PRP8* (L*er*) vs *prp8* (*sof81*) Student's t-test showed P-values < 0.05.

**Figure S3. *prp8-6* does not disrupt FCA-feedback regulation and recruitment to *FLC*, related to Figure 3.**

(A) Northern analysis of transcripts derived from the 35S-*FCAγ* transgene. Total RNA was hybridised with a probe for 35S-*FCAγ*. Two major transcript species (indicated on left, read-through transcripts (Liu et al., 2012) indicated with *) are detected in C2 and *prp8-6*, none in the non-transgenic L*er*. (B) Analysis of *FCA*-auto feedback regulation. Protein extracts were analysed by western blotting and probed with FCA specific antibodies (Macknight et al., 1997). The highly abundant protein present in C2 and *C2 prp8-6* is the protein derived from the *35S-FCA*γ transgene (Quesada et al., 2003). The less abundant species with higher molecular weight detected in L*er* represents the endogenous protein; it is 10 kD longer than the transgenic protein due to a non-canonical translational start codon (Simpson et al., 2010). In C2, expression of the endogenous *FCA* is repressed by *35S-FCA*γ. The lack of endogenous FCA protein in C2 *prp8-6* shows the mutant does not disrupt FCA-feedback regulation. (C) Analysis of *FCA*-auto feedback regulation by *FCA* transcript analysis. Total RNA was hybridized with a *FCA* 5'UTR probe that specifically detects the endogenous *FCA* transcripts. Three *FCA* transcript species (indicated on left) are detected in L*er*, but only *FCAβ* is detected in C2 and *C2 prp8-6*. This indicates that *FCA*-auto feedback regulation is not sensitive to the *prp8-6* mutation. *APT* is shown as loading control (below). (D) Schematic representation of *FLC* with black rectangles denoting exons. Horizontal bars (A, C, G and H) denote regions analyzed by qPCR in chromatin immunoprecipitation (ChIP) experiments. FCA association with different *FLC* regions; ChIP assay of *prp8-6* (grey), each region normalized to C2 and calculated as the % input normalized to binding to *Actin*. Values represent means +/- SEM (n =

3). Student's t-test was performed, P-values < 0.05 for enrichment of FCA at G in *prp8-6* relative to wild type. (E) Relative expression of *FPA*, *FLD* and *LD* in Ler and *prp8-6*. 5 µg of total RNA from seedlings were reverse transcribed using a mixture of reverse primers corresponding to *FPA* (FPAex2R), *FLD* (FLDex3R) and *LD* (LD3R). Primers across the splice junctions of the intron 1 were used to measure the expression of FPA (FPA1-2F and FPAex2R). Primers across the splice junction of the intron 2 were used to measure the expression *FLD* (FLDex2-3F and FLDex3R) and *LD* (LD3F and LD3R). The expression of *FPA*, *FLD* and *LD* was normalized to *UBC*, and error bars represent SEM (n = 3). No significant differences were found at P < 0.05.

**Figure S4. *FLC* expression compared to the splicing *cis*-element mutant *flc-5* and *COOLAIR* expression by northern blotting, related to Figure 3.** (A) Northern analysis of *FLC* transcripts in *prp8-6* and *flc-5*. Two biological repeats are analysed, the experiment compares sense *FLC* transcripts in the presence of *prp8-6*, a *FLC* sense splice site *cis*-element mutant (*flc-5*) and L*er* wild-type. In *flc-5* the splice acceptor AG dinucleotide of *FLC* intron 2 is changed to AA (Greb et al, 2007). *APT* is shown below as loading control. High molecular weight RNA species of low abundance were detected in *flc-5* but no additional transcripts were detected in *prp8-6* or L*er*. (B) *COOLAIR* Class Ii intron1 splicing efficiency in *prp39* was not significantly different to Col-0 at P < 0.05. The splicing efficiency was measured by qPCR as described in the material and methods. Values represent biological means +/- SEM (n = 3). (C) Northern analysis of *COOLAIR* transcripts in *FRI, FRI fca-1* and *FRI prp8-6*. 40µg total RNA was loaded to detect low abundance. The blot was probed with an antisense specific single strand probe generated using primers that will hybridize to all *COOLAIR* transcripts. The two major transcript types previously characterized – class I and class II can be seen – very low abundance plus alternative splicing and heterogeneity of the exact poly (A) site contribute to the fuzziness of the hybridizing fragments. The ratio of proximal and distal polyadenylated transcripts was calculated for the analysed genotypes and displayed below to represent the relative reduction in class I in *prp8-6.* Values represent means of four biological repeat RNA preparations +/- SEM. Student's t-test showed statistically significant differences of *FRI* vs *FRI*/*fca* and *FRI* vs *FRI*/*prp8* with P-values < 0.05.

**Figure S5. Design and analysis of *FLC$^{tex}$*, related to Figure 4.** (A) Schematic representation of the *flc-2* mutant fast-neutron-induced allele (Michaels and Amasino 2001). Red indicates the genomic sequence missing in *flc-2* allele. (B) Schematic representation of the *FLC$^{tex}$* transgene. The *FLC$^{tex}$* or wild-type *FLC* transgene were transformed into *FRI flc-2* and independent lines identified.

(C) Levels of proximally polyadenylated antisense transcript were measured by RT-qPCR in *FLC-TEX* lines (QS549, #573 and #577) and controls. gFLC-v3 is a representative transgenic line carrying a wild-type *FLC* genomic clone described in (Coustham et al., 2012), the wild-type Col-0 and the *flc-2* mutant are non-transformed reference controls. Values represent means of three biological repeats +/- SEM.

**Figure S6. Analysis of cryptic splicing in *COOLAIR*$^{AA}$ and additional representations of ChIP data, related to Figures 5 and 6.** (A) Mutation of the natural 3' splice site of the Class Ii *COOLAIR* intron does not lead to use of nearby cryptic 3' splice sites. The schematic representation of the *COOLAIR*$^{AA}$ mutation is shown with primers flanking the Class Ii intron. P1 and UBC-R were used for reverse transcription (RT) and P1 with P2 for amplification. The PCR products were analyzed by agarose gel electrophoresis. The PCR fragment from the wild-type spliced transcript is absent in *COOLAIR*$^{AA}$, and no novel PCR fragments are detected in *COOLAIR*$^{AA}$. UBC amplification is shown as loading control; no amplification was observed in the no RT control. (B-D) Additional representations of the Pol II and H3K4me2 ChIP experiments shown in Figure 6A-B. (B) Representations of H3K4me2 ChIP data. Four independent ChIP experiments comparing the C2 control (black) to *prp8-6* (grey) were performed. Data is represented as % of input chromatin normalized to H3K4me2 at the Actin gene used as internal control. Bar graph shows the mean of the four experiments at each *FLC* region in (Figure S3D), error bars represent +/- SEM. (C) Representations of RNA Pol II ChIP data. Four independent ChIP experiments comparing the C2 control (black) to *prp8-6* (grey) were performed. Data is represented as % of input chromatin normalized to Pol II binding to the *Actin* gene used as internal control. Bar graph shows the mean of the four individual experiments at each *FLC* region as in (Figure S3D), error bars represent +/- SEM. (D) Technical controls for ChIP experiments. One representative sample dataset (Pol II ChIP in C2 background of one experiment) is shown. Pol II binding is represented as % of chromatin input, values of IP against Pol II are given in black (Ab) and values of no antibody controls (always performed for each sample in each experiment) are given in red on the right side of the IP bars (no Ab). As values obtained for the no antibody are much lower than those for IP against Pol II values, they are inserted into the graph. Note how Pol II binding to the constitutively transcribed *ACTIN* gene is higher than to any *FLC* region.

**Figure S7. Synergistic genetic interaction between weak *fld* and *prp8* alleles and analysis of *tfIIs* mutants, related to Figure 6.** (A) The *fld-6* and *prp8-6* single mutants are compared to the *fld-6/prp8-6* double mutant and the C2 background control. The FLC-LUC fusion transcript is

larger than the endogenous *FLC* transcript and marked (*). *APT* is shown as loading control. (B-D) *Arabidopsis* TFIIS does not appear to be required for *FLC* repression and proximal polyadenylation of *COOLAIR*. *FLC* transcription was analysed in *Arabidopsis tfIIs* mutants. (B) Analysis of sense *FLC* levels by RT-qPCR normalized to the *UBC* control. (C) Analysis of total *COOLAIR* transcription levels normalized to *UBC*. (D) Determining the fraction of *COOLAIR* transcripts polyadenylated at the proximal position. RT-qPCR data are means derived from three biological repeats +/- SEM. None of the means are statistically significant at $P < 0.05$.

## Table S1: Oligonucleotides used in study, related to data analysis in all figures.

| Name | Sequence(5’ to 3’) |
|---|---|
| Int1_RT | CTGCTGGACAAATCTCCGACAATC |
| Int1_spliced_LP | GACAAATCTCCGACAATCTTCC |
| Int1_spliced_RP | CTCACACGAATAAGGTGGCTAAT |
| Int1_unspliced_LP | CGACAATCTTCCGGTGACTCT |
| Int1_unspliced_RP | TACAAACGCTCGCCCTTATC |
| class II-2_LP | CTCCTCCGGCGATAAGTA |
| Class II-1_LP | CTCCTCCGGCGATAAGTA |
| Class II-1_RP | CTCACACGAATAAGAAAAGTAAAA |
| Class II-2_RP | ACGATAATCATAGAAAAGTAAAAGAGC |
| Class II-3_RP | TTGTCCAGCAGAAAAGTAAAAGA |
| Class II-4_LP | CCGGCGGATCTCTTGTTGT |
| Class II-4_ RP | AATTCTCACACGAATAAGAAAAAAACAC |
| Class II-4_int2_RP | AAAAACACAAACAAACACAGAACC |
| Class II unspliced F | TCGCTCTTCTCGTCGTCTC |
| Class II unspliced R | AAAACACAAACAAACACAGAACC |
| FLC Unspliced_LP | CGC AATTTTCATAGCCCTTG |
| FLC Unspliced_RP | CTTTGTAATCAAAGGTGGAGAGC |
| FLC Spliced_LP | AGCCAAGAAGACCGAACTCA |
| FLC Spliced_RP | TTTGTCCAGCAGGTGACATC |
| Total COOLAIR_LP | TGTATGTGTTCTTCACTTCTGTCAA |
| Total COOLAIR_RP | GCCGTAGGCTTCTTCACTGT |
| FLC3ss_F1-forward | TTTTATTGTACATCAGATATATCCTCTTCTGTGTTG |
| FLC3ss_F1-reverse | GCCGATTTAAAGTGGCTAATTAAGTAGTGG |
| FLC3ss_F2-forward | CTCTCCCACTACTTAATTAGCCACTTTAAA |
| FLC3ss_F2- reverse | TGTGAAGCAAACACAAGTTTTTGACAG |
| set1_RP | CACACCACCAAATAACAACCA |
| LP_FLCin6polyA | TTTTTTTTTTTTTTTACTGCTTCCA |
| UBC-F | CTGCGACTCAGGGAATCTTCTAA |
| UBC-R | TTGTGCCATTGAATTGAACCC |
| UBC9.1-intron1-SF | CGTGAATTCGGAAGTCTTCAA |
| UBC9.1-intron1-SR | GCGCTACATGAAGTAGGAGGA |
| UBC9.1-intron1-UF | TTTGGATCTTCTTCCCGTCTT |
| UBC9.1-intron1-UR | AATCCCACGATCCAAATTCC |
| EF1a-intron3-SR | TCCTTCTTGTCCACGCTCTT |
| EF1a-intron3-SF | GATTGAGAAGGAGCCCAACC |
| EF1a-intron3-UF | ATGGTGACGCTGGTATGGTT |
| FLC.1-SF | GGCTAGCCAGATGGAGAATAA |
| FLC.1-SR | TCAACCGCCGATTTAAGGT |
| FLC.1-UF | TGGTTGTTATTTGGTGGTGTG |
| FLC.1-UR | GGAGAGTCACCGGAAGATTG |
| FLC.2-SR | TGTACGATAATCATAGGTCAAAATCA |
| FLC.2-SF | AAAATGCTGAAAGAAGAGAACCA |
| FLC.2 U F | TGGTTGTTATTTGGTGGTGTG |
| FLC.2 UR | TCTCCATCTCAGCTTCTGCTC |
| FLC.3-SF | AAATGCTGAAAGAAGAGAACCAG |
| FLC.3-SR | ACTTCTAGACACTTGGAGTTGGA |
| FLC.3-UF | TTGGTTTCCTTGAAGGTTGTG |
| FLC.3-UR | ACTTCTAGACACTTGGAGTTGGA |
| FLC.4-SF | TGTTGAGAATCTTAAAGAAAAGATGG |
| FLC.4-SR | GGAGAGTCACCGGAAGATTG |
| Class I_LP | ACTGCTTCCAAACTTAAAAGC |
| Set4_LP | TTTTTTTTTTTTTTTGCGGTACAC |
| Set4_RP | GGGGTAAACGAGAGTGATGC |
| LB3 | TAGCATCTGAATTTCATAACCAATCTCGATACAC |
| PRP8_3’1 | AGAGACTTGTATTGCTTGTTCTTCCA |

| | |
|---|---|
| PRP8_6600F | ACCTTAGAGTCAATCACATATATGTGA |
| PRP8_900R | TCCAATTGAATTGGCTCCAGA |
| 81_BglII_cCAPS_F | ACTACTTCTGTTTCTCTAGTAACAGGCTACA |
| 81_BglII_dCAPS_R | AGCAAGTGAACCTCAAGGGGATCCAAGATC |
| 81 MboII dCAPS F | CAAAGCAGATTATTGTCACACGGGAAG |
| 81 MboII dCAPS R | GGCAAGCCTGGAACGGAAGC |
| flc-2F | GTCAAAACTCAAGCCTCAAAACC |
| Flc-2R | GCGTCGTGGAAGATGTGTAACTC |
| FCAgaF3 | TGGTCATGTGGAAGATGTCTATCTCA |
| FCAgaR3 | ACGAGGCCCTGAACCAAGTCCA |
| PRP8-SacII-SbfI-F | TCCCCGCGGCCTGCAGGTTCTTCAGCCTTCAGGTG ACTGT |
| PRP8-KpnI-R | CCGGTACCTAGAGGAGGGATGATAAAACTGCTGT |
| FLCexon7.5’ | GGAGAATAATCATCATGTGGGAGCA |
| FLC3UTR.3’ | CTCACACGAATAAGGTACAAAGTTC |
| FCA 5lead5' | GGGCTCCTAGTCCTTTGATTTCTT |
| FCA 5lead3' | TTGCTAGGGCTGCTTCCACGAC |
| FCA.E2.F | CTTTGGTGACTCTAGTGGGGATGT |
| FCA FR | GGAGACCCTGTGGACTGTGAAGAG |
| FPA1-2F | ATACGCACGACCGGCAAAAC |
| FPAex2R | AAAGCTGTCTTGCGTTCTCT |
| FLDex2-3F | GGCACAAAATCTGGTATATT |
| FLDex3R | CTGATCATCTTCCGCTTCAA |
| LD3F | TCCATTAAAATTGGAAAGCGG |
| LD3R | TACTAATAGCATCTTTAATTG |

## Extended Experimental Procedures

**Genetic materials**: The parental C2 line was generated by crossing L*er* containing the *35S::FCAγ* transgene, (over-expressing *FCA*) (Macknight et al., 1997), to L*er* containing transgenic *FRI* and *FLC-LUC* (Liu et al., 2007). We ensured that the three transgenes in C2 were homozygous before using the line for mutagenesis. A line (gvF) was generated in the Col background containing the same three transgenes, at the same genomic location, by crossing and then backcrossing the C2 parental line nine times to the Col wild type (Liu et al., 2010). The three transgenes were confirmed as homozygous before using it for genetic mapping of *sof81.* The *prp8* T-DNA mutant alleles *sus2-4* (ABRC stock: CS16072) and *sus2-5* (ABRC stock: CS16073) were ordered from the *Arabidopsis* stock centre (NASC) and propagated as heterozygotes. *prp8-6* was generated by crossing *sof81* to L*er*, the resulting F2 population was screened for individuals homozygous for *prp8-6* and the absence of *FRI (JU223)* and *35S::FCAγ. FRI/prp8-6* was identified from the same F2 generation; however individuals homozygous for *prp8-6* in the presence of *FRI (JU223)* but in the absence of *35S::FCAγ* were isolated. Genotyping and segregation for early flowering individuals in the F3 populations of these individuals revealed which lines were homozygous for *FRI (JU223)*. *FRI/prp8-6/fca-1* was generated by crossing *sof81* to a *prp8-6/fca-1* double mutant isolated from a cross between *prp8-6* and *fca-1*. This enabled screening the resulting F2 generation for individuals

homozygous for *fca-1* in the presence of *FRI (JU223)* but in the absence of *35S::FCAγ* to isolate the *FRI/prp8-6/fca-1* genotype. The *sof1/sof81* double mutant was generated by crossing the *sof1* mutant (Liu et al., 2007) with *sof81* and double homozygous individuals identified by genotyping.

The flowering time mutants *fca-1*, *fca-9*, *flc-5, fld-4, fld-6, flc-2* used in this study have been previously described (Greb et al., 2007; Liu et al., 2010; Liu et al., 2007; Macknight et al., 1997; Manzano et al., 2009). *tfIIs* mutants are described in (Grasser et al., 2009). *prp39* mutants are described in (Wang et al., 2007).

**Mutagenesis and screen of *sof* mutants**: Chemical mutagenesis of the parental line C2 was carried out as described in (Liu et al., 2007). *sof* mutants were identified in the resulting M2 population by screening for seedlings with increased FLC-LUC bioluminescence activity compared to the parental C2 control line, using the light sensitive Photek and Nightowl CCD camera systems (Liu et al., 2010).

**Flowering time analysis**: plants were grown in controlled environment rooms with a photoperiod of 16 hours light and 8 hours dark. Temperature ranged between 23-25 °C during the day and 20-22 °C at night. Rosette leaf number (RLN) produced by the main apical meristem before switching the developmental program to the initiation flowering was counted to measure variation in flowering time.

**FLC-LUC detection**: seedlings around 10-15 days after germination on plates of GM medium or soil were sprayed with 1 mM of luciferin (Promega) substrate solution and incubated in the dark at room temperature for 20 minutes. LUC bioluminescence activity of the seedlings was assayed using either the Photek or Nightowl light sensitive CCD camera detection systems. Light emitted by the FLC-LUC reporter was captured and quantified. For the purpose of representation photon counts per pixel were false coloured and superimposed on a photograph of the analysed *Arabidopsis* seedlings. Warm colours (yellow-red) represent a high FLC-LUC bioluminescence activity and cold colours (blue) represent a low FLC-LUC bioluminescence activity. A threshold of the displayed counts was chosen so that FLC-LUC bioluminescence activity of known low *FLC* expressing genotypes, always assayed alongside as internal negative controls, was only just detectable.

**Genotyping**:

*sus2-4* was genotyped with the T-DNA left border specific oligonocleotide LB3 and PRP8_3'1 to detect the presence of the insertion. PCR using PRP8_6600F and PRP8_3'1 tested whether the T-DNA insertion was homozygous.

*sus2-5* was genotyped with the T-DNA left border specific oligonucleotide LB3 and PRP8_900R to detect the presence of the insertion. PCR using PRP8_600F and PRP8_900R tested whether the T-DNA insertion was homozygous.

*prp8-6* was genotyped by dCAPS marker with the oligonucleotides 81_BglII_cCAPS_F and 81_BglII_cCAPS_R followed by digestion of the PCR products with BglII and 4% agarose gel electrophoresis. BglII cleaves the mutant sequence (S1B).

An alternative method for *sof81* genotyping was using a dCAPS marker with the forward primer sof81-MboII-dCAPS-F and reverse primer sof81-MboII-dCAPS-R. Digestion with MboII resulted in 25 bp and 92 bp fragments if *sof81* was present.

*flc-2* genotyping was performed with forward primer flc2-F and reverse primer flc2-R. These primers amplify the fragment that is located within the deleted region of the *flc-2*.

*35S::FCAγ* was genotyped by using the oligonucleotides FCAgaF3 and FCAgaR3 Presence of *35S::FCAγ* was detected by amplification of a ~200 bp product from *FCAγ* cDNA template in addition to a ~500 bp product resulting from amplification of genomic *FCA*.

**Genetic mapping of *sof81***: *sof81* was backcrossed twice to the parental line to reduce the presence of interfering secondary mutations. Backcrossed *sof81* was then crossed to a Columbia line (containing the same transgenes) and the F2 screened for FLC-LUC bioluminescence. Plants with high FLC-LUC bioluminescence activity in the resulting F2 were selected and genotyped using molecular markers polymorphic between the L*er* and Col. Candidate genes lying within the final mapping interval were amplified and sequenced in the parental line as well as in the *sof81* mutants to identify specific base changes. We identified that *sof81* carries G to A transition consistent with EMS mutagenesis at position 7585 bp in the genomic sequence of *Arabidopsis PRP8* (starting the ORF-ATG). The G to A transition in *sof81* changes the GGA coding for glycine at position G1891 in the *PRP8* ORF to GAA coding for glutamic acid instead (see also Fig. 1).

**Northern blot analysis**: A *FLC* cDNA fragment, including the last exon and 3' UTR, was amplified with primers FLCexon7.5' and FLC3UTR.3'. The purified PCR product was labelled (with $\alpha$-P$^{32}$ dCTP) by a primer extension reaction, using primer FLC3UTR.3', to generate a single-stranded antisense probe to detect *FLC* or *FLC-LUC* on total RNA blots.

The probe to analyze *FCA* feedback regulation by northern blotting was generated by first amplifying a PCR product using FCA 5lead5' and FCA 5lead3' using genomic DNA as template. The purified PCR product was labelled (with $\alpha$-P$^{32}$ dCTP) by a primer extension reaction, using the 5lead3' primer, to generate a single-stranded antisense probe to specifically detect endogenous *FCA* sense transcript species on total RNA blots.

The *COOLAIR* northern was generated using 40μg total RNA. The blot was probed with an antisense specific single strand probe generated using primers Total COOLAIR_LP and RP to amplify the DNA and Total COOLAIR_LP to synthesize the labelled probe. This probe will hybridize to all *COOLAIR* transcripts.

The probe to analyze expression of the *35S::FCAγ* transgene by northern blotting was generated by first amplifying a PCR product using fca.e2.f and FCA FR. The purified PCR product was labelled (with $\alpha$-P$^{32}$ dCTP) by a primer extension reaction, using the FCA Fr primer, to generate a single-stranded antisense probe to specifically detect transgenic *FCAγ* sense transcript species on total RNA blots. Hybridizations were performed using Ultrahyb buffer (Ambion), following the manufacturer's instructions. The blots were stripped and re-probed with loading controls such as *APT* (Marquardt et al., 2006).

**Western blot analysis** to detect FCA using the KL4 antibody was performed as described (Manzano et al., 2009; Quesada et al., 2003). Slight modifications were implemented for the detection of *Arabidopsis* PRP8 using the BMR-00434 antibody. Blocking was performed in 3% BSA 1xTBS and antibody incubation in 1.5% BSA 1xTBS.

**Chromatin immunoprecipitation** (ChIP) was performed as described (Liu et al., 2007) using H3K4me2 antibody (Upstate, 07-030), Pol II antibody (Abcam, ab817) and anti-FCA antibody (KL4). ChIP-qPCR primers used to assay *FLC* regions are shown in Supplemental table 1. Variation in chromatin amount was accounted for by normalizing the qPCR data of IP and no antibody controls to the signal from 10% of the input chromatin (representing it as % of input). To account for differences in IP and crosslinking efficiencies between samples, the data were normalized to enrichment of the Actin locus, co-assayed as internal control (representing data as %

input/*Actin*). Four independent ChIP experiments were performed and values for each region in each experiment are derived from at least three technical repeats. Data using the %input/Actin analysis method are supplied in Figure S6, data normalized to the control genotype is used to visualize regions with greatest relative change in *prp8-6* in Figure 6.

**Measuring splicing efficiency in *UBC9, EF1a* and sense *FLC*.**

We measured splicing efficiency of the alternative spliced introns of *UBC9* (*At4G27960.1* intron 1) and *EF1a* (*At5g60390.2* intron 3). 5 μg of total RNA of L*er FRI* or L*er FRI prp8-6* were reverse transcribed with primers UBC9.1-intron1-SR and UBC9.1-intron1-UR that are located in the exon 2 of *At4G27960.1*, and EF1a-intron3-SR that is located in exon 4 of *At5g60390.2*. The resulting cDNA was used as template for quantitative RT-PCR reactions to measure the spliced and unspliced transcript levels. UBC9.1-intron1-UF and UBC9.1-intron1-UR were employed to measure the unspliced intron 1 of *At4G27960.1*. UBC9.1-intron1-SF and UBC9.1-intron1-SR were employed to measure the splicing junction of exon 1-exon 2 of *At4G27960.1*. EF1a-intron3-UF and EF1a-intron3-SR were employed to measure the unspliced intron 3 of *At5g60390.2*. EF1a-intron3-SFand EF1a-intron3-SR employed to measure the splicing junction of exon 3- exon-4 of *At5g60390.2*.

In order to measure the alternative splicing of sense *FLC*, 5 μg of total RNA were primed by using a mixture of primers that are listed below to specifically reverse-transcribe different forms of spliced and unspliced *FLC* sense. Primers used in the reverse transcription: FLC.1-SR located in the exon 7 of *FLC.1*; FLC.2-UR located in the exon 7 of *FLC.2*; FLC.3-SR located in exon 7 of *FLC.3*; FLC.4-SR located in the exon 6 of *FLC.4*. The primers used for the qPCR amplification are listed below. FLC.1-SF and FLC.1-SR were employed to measure the splicing junction of exon 6-exon 7 of *FLC.1*. FLC.2-SF and FLC.2-SR were employed to measure the splicing junction of exon 6 - exon 7 of *FLC.2*. FLC.3-SF and FLC.3-SR were employed to measure the splicing junction of exon 6 - exon 7 of *FLC.3*. FLC.4-SF and FLC.4-SR were employed to measure the splicing junction of exon 5 - exon 6 of *FLC.4*. FLC.1-UF and FLC.1-UR were employed to measure the unspliced intron 6 of FLC.1 and unspliced intron5 of FLC.4. FLC.2-UF and FLC.2-UR were employed to measure the unspliced intron 6 of FLC.2. FLC.3-UF and FLC.3-UR were employed to measure the unspliced intron 6 of FLC.3. Triplicates of all PCR reactions were performed and quantified against standard curves before calculating the mean. The spliced/unspliced ratio was then calculated and

normalized to the control genotype. Averages and standard errors of the spliced/unspliced ratios were calculated from data of three biological replicates for each.

**Statistical analysis**

We performed paired sample t-tests and denoted statistically significant differences with $p < 0.05$ in the figures throughout the manuscript by a (*), or noted the result of statistical testing in the figure legend.

## Supplemental References


Manzano, D., Marquardt, S., Jones, A.M.E., Bäurle, I., Liu, F.and Dean, C. (2009) Altered interactions within FY/AtCPSF complexes required for Arabidopsis FCA-mediated chromatin silencing. Proc. Natl. Acad. Sci USA *106*, 8772-8777.

Marquardt, S., Boss, P.K., Hadfield, J., Dean, C. (2006) Additional targets of the Arabidopsis autonomous pathway members, FCA and FY. J Exp Bot. *57*, 3379-86.

Schmid, M., Davison, T.S., Henz, S.R., Pape, U.J., Demar, M., Vingron, M., Schölkopf, B., Weigel, D., Lohmann, J.U. (2005) A gene expression map of *Arabidopsis thaliana* development. Nat Genet. *37*, 501-506.

# Supplemental figure 1

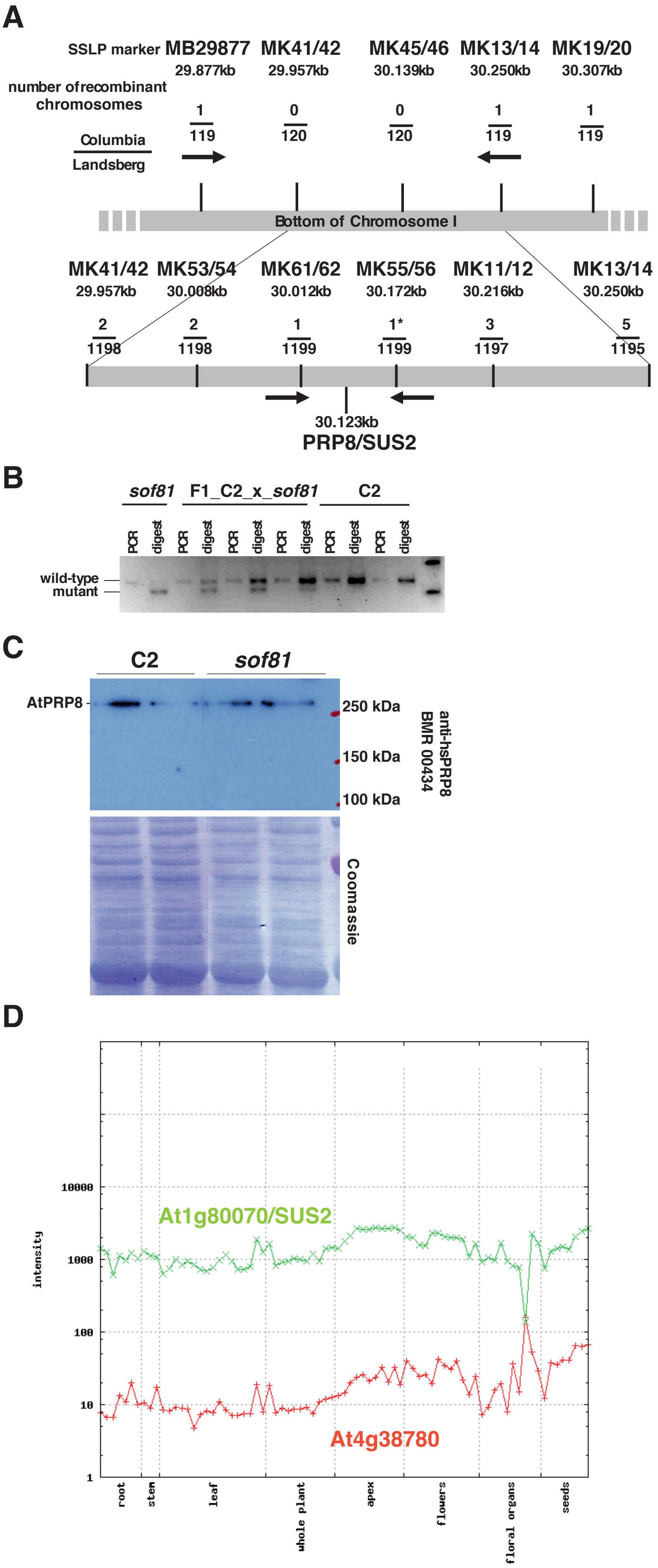

# Supplemental figure 2

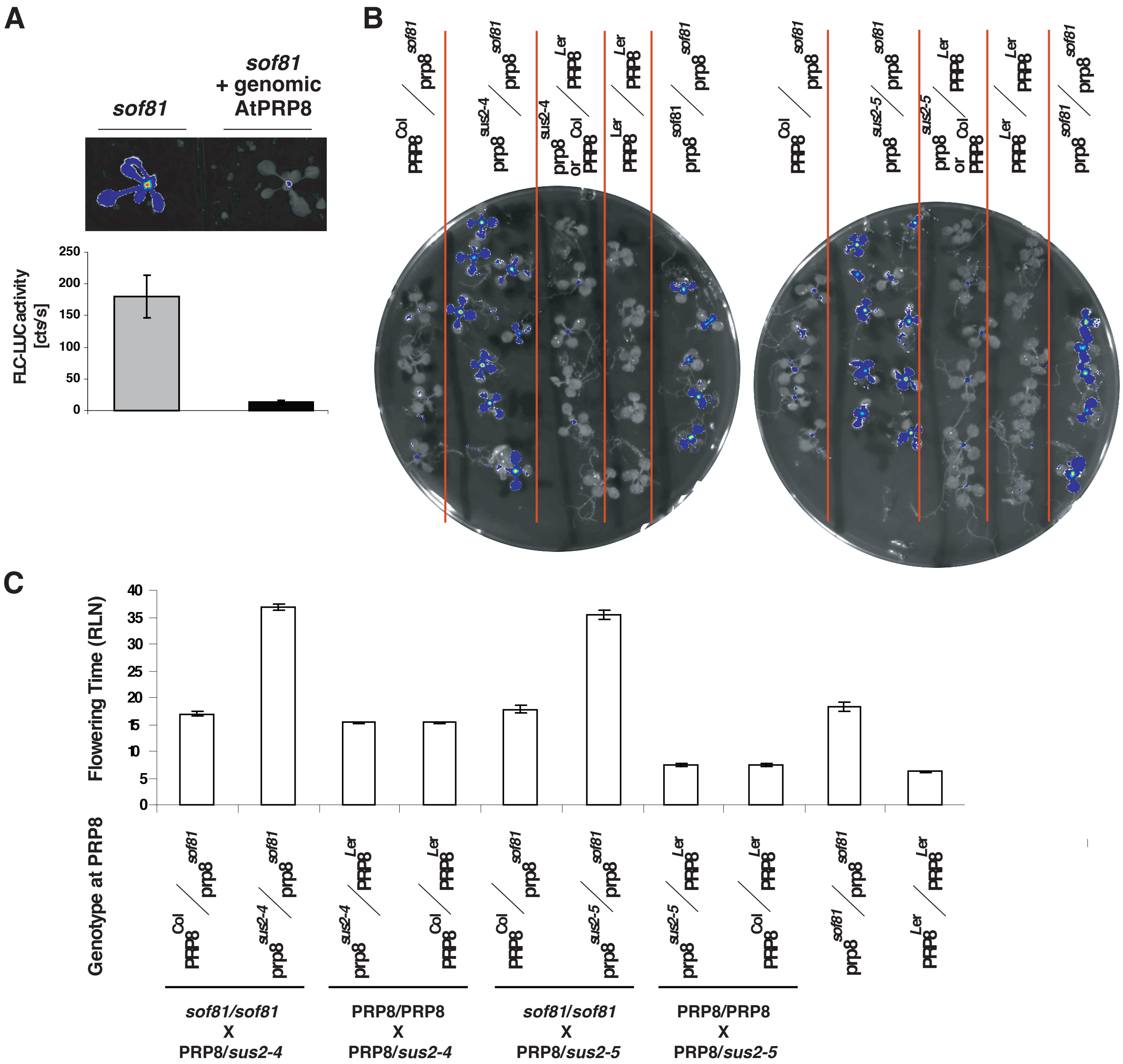

# Supplemental figure 3

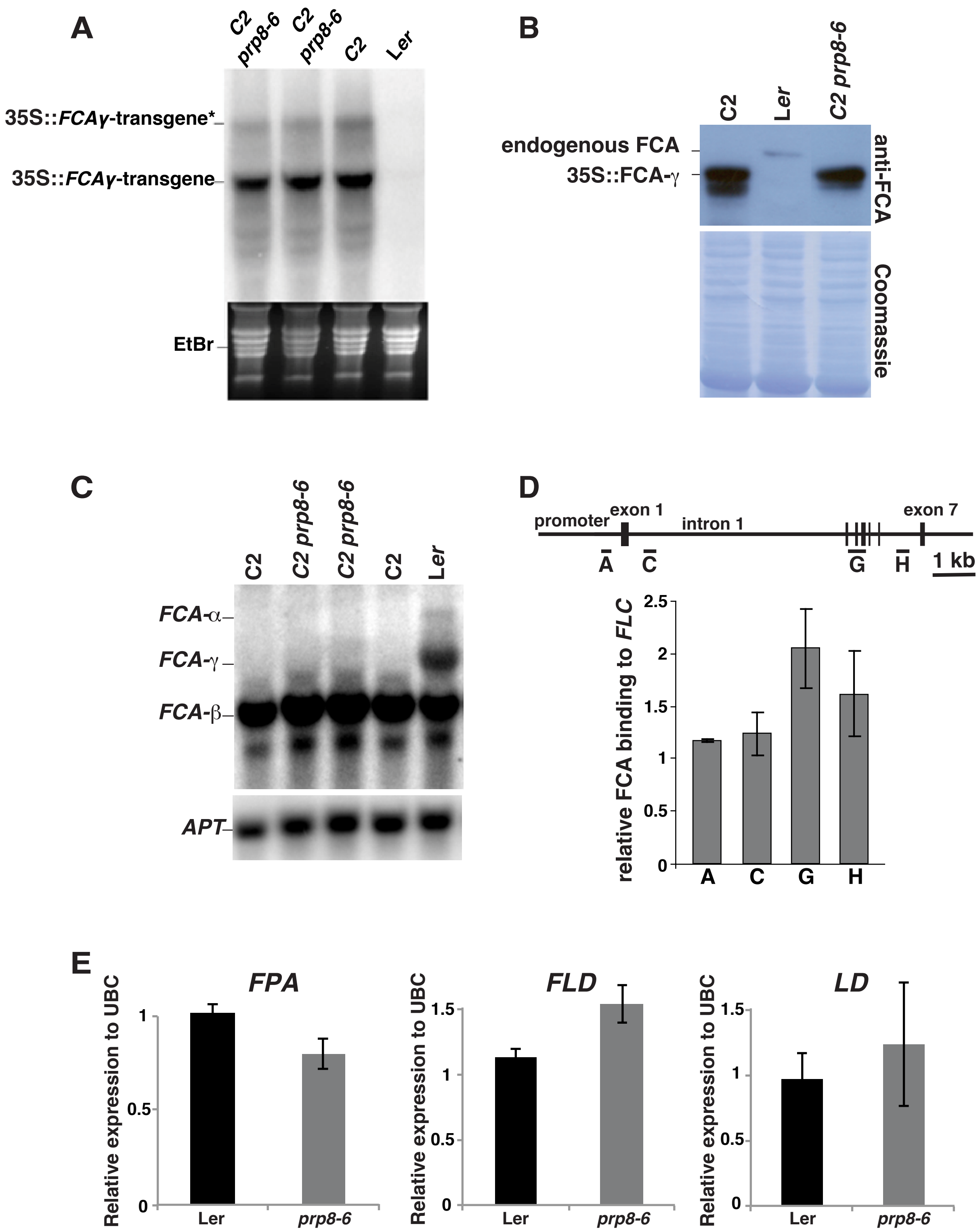

# Supplemental figure 4

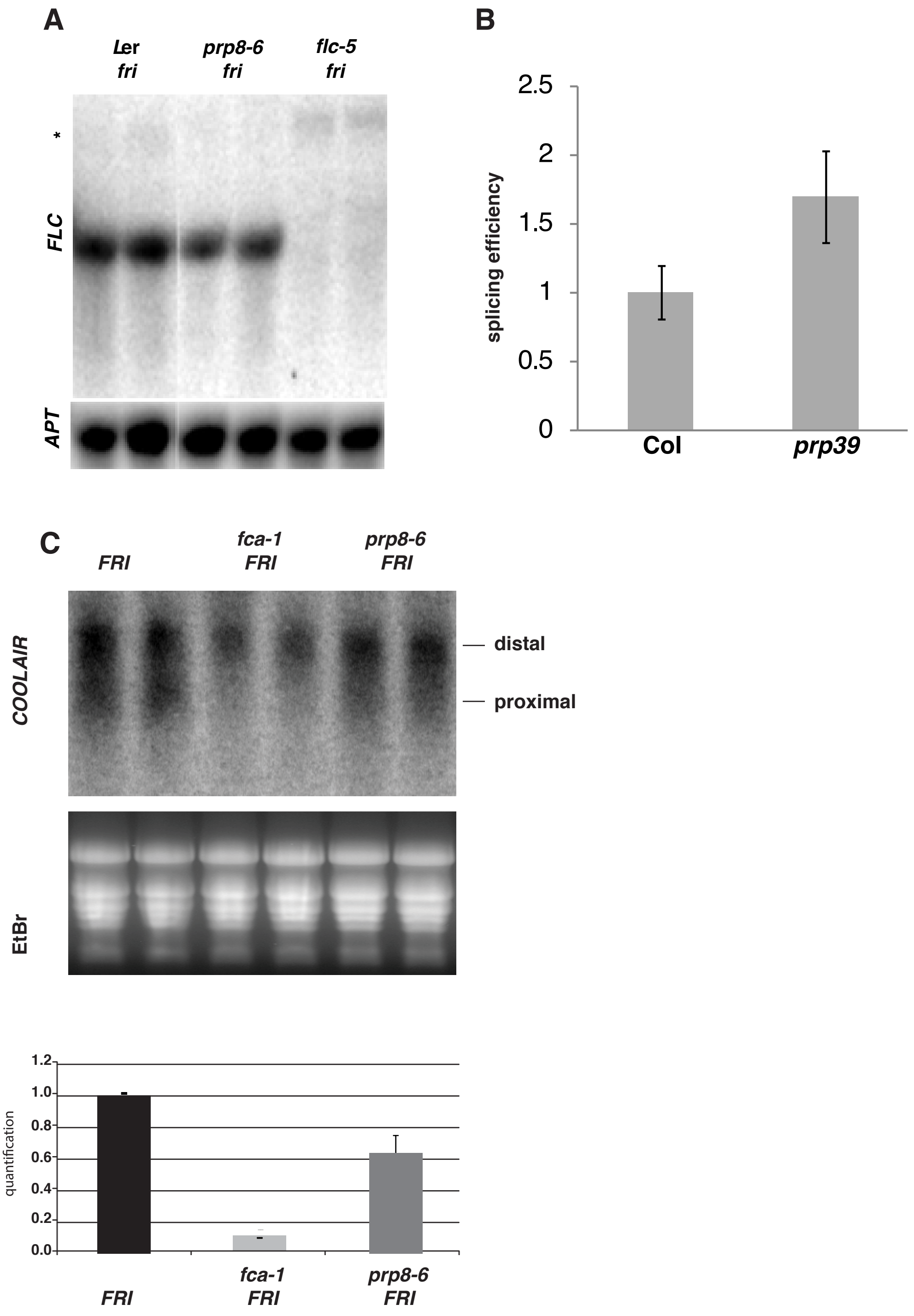

# Supplemental figure 5

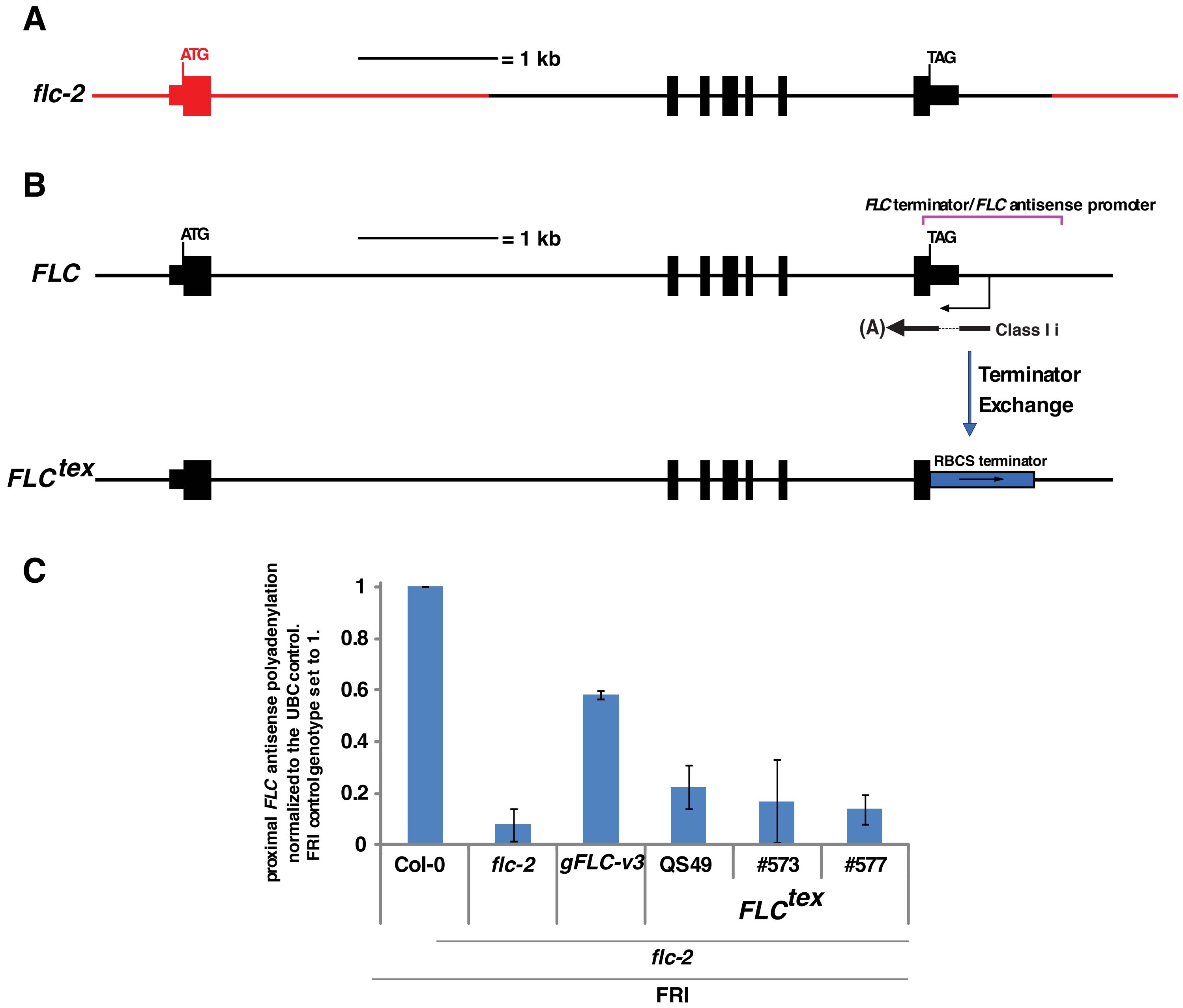

# Supplemental figure 6

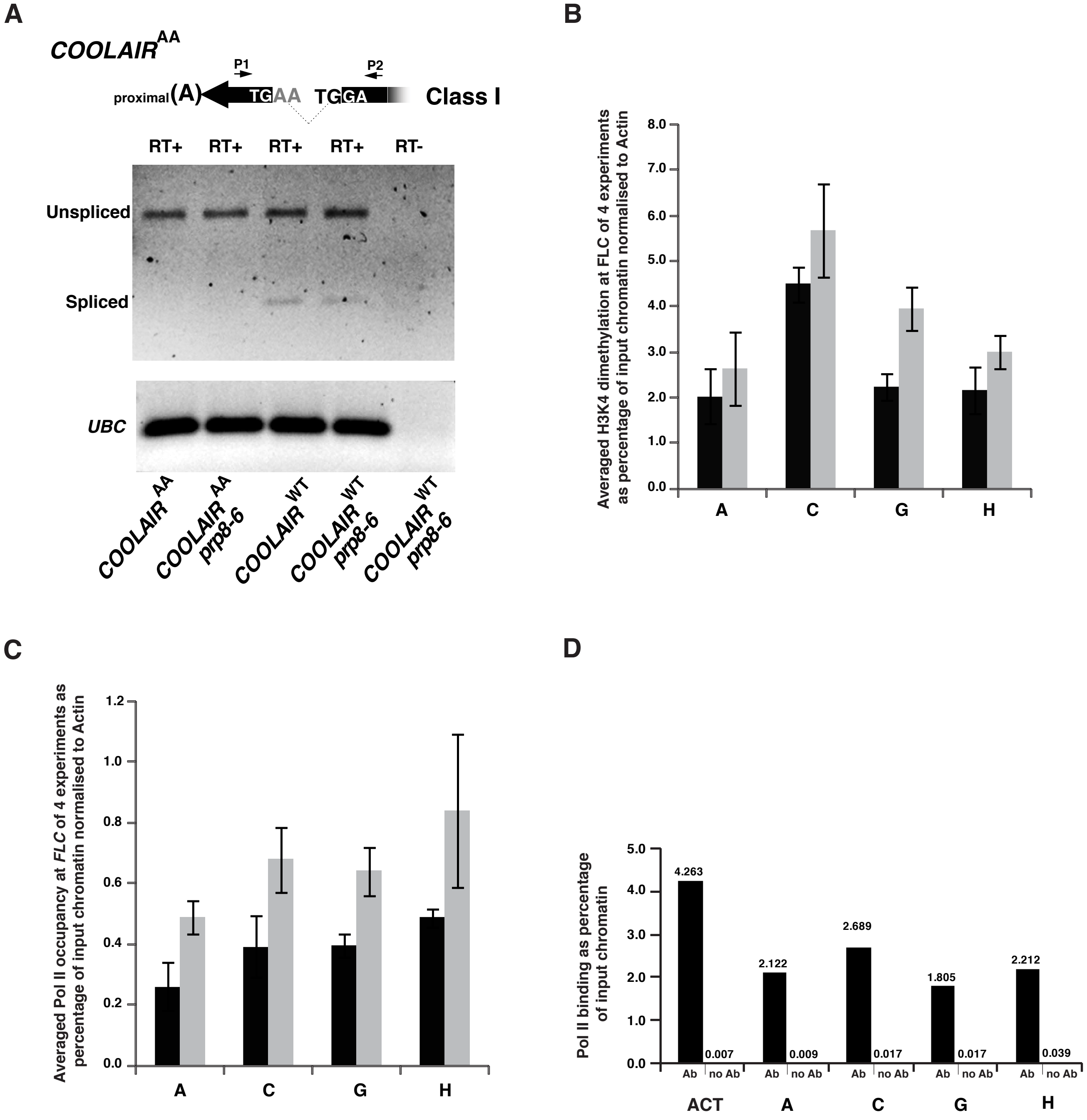

# Supplemental figure 7

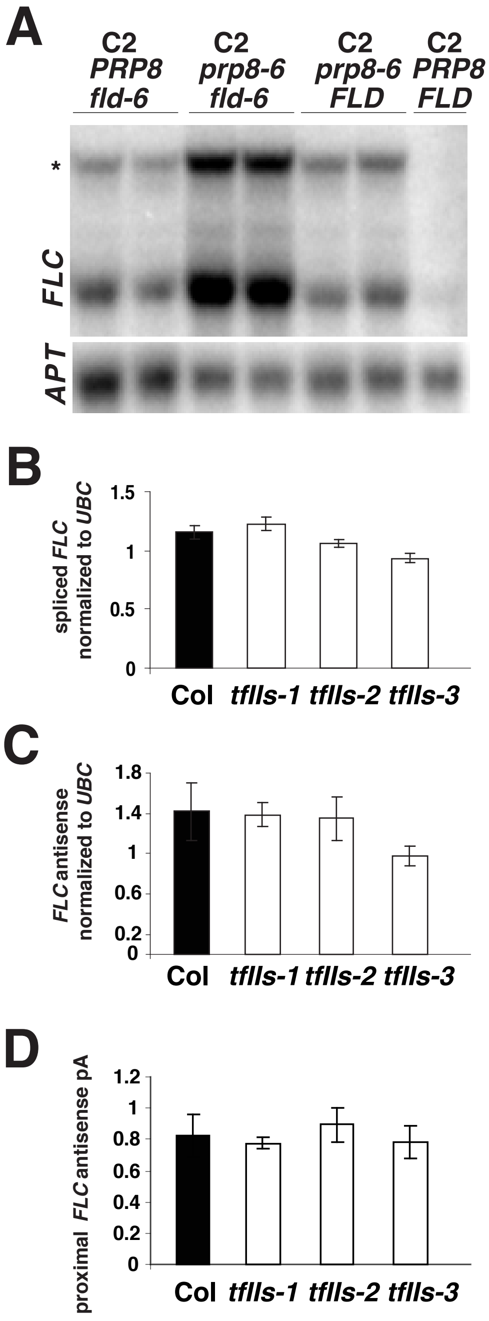

Supplement: Document S1. Figures S1–S7, Table S1, and Supplemental Experimental Procedures [file mmc1.pdf]
