# Supplementary material for: Functional Consequences of Splicing of the Antisense Transcript COOLAIR on FLC Transcription
Source: Mol Cell. 2014 Apr 10;54(1):156–65. doi: 10.1016/j.molcel.2014.03.026 (PMC3988885; doi:10.1016/j.molcel.2014.03.026)

Molecular Cell
Article

Functional Consequences of Splicing of the Antisense Transcript *COOLAIR* on *FLC* Transcription

Sebastian Marquardt,[1,2] Oleg Raitskin,[1] Zhe Wu,[1] Fuquan Liu,[1,3] Qianwen Sun,[1] and Caroline Dean[1,*]
[1]Department of Cell & Developmental Biology, John Innes Centre, Norwich Research Park, Norwich NR4 7UH, UK
[2]Present address: Department of Biological Chemistry and Molecular Pharmacology, Harvard Medical School, Boston, MA 02115, USA
[3]Present address: Institute of Global Food Security, School of Biological Sciences, Queen's University, Belfast, BT9 7BL, UK
*Correspondence: caroline.dean@jic.ac.uk
http://dx.doi.org/10.1016/j.molcel.2014.03.026
This is an open access article under the CC BY license (http://creativecommons.org/licenses/by/3.0/).

SUMMARY

Antisense transcription is widespread in many genomes; however, how much is functional is hotly debated. We are investigating functionality of a set of long noncoding antisense transcripts, collectively called *COOLAIR,* produced at *Arabidopsis FLOWERING LOCUS C* (*FLC*). *COOLAIR* initiates just downstream of the major sense transcript poly(A) site and terminates either early or extends into the *FLC* promoter region. We now show that splicing of *COOLAIR* is functionally important. This was revealed through analysis of a hypomorphic mutation in the core spliceosome component PRP8. The *prp8* mutation perturbs a cotranscriptional feedback mechanism linking *COOLAIR* processing to *FLC* gene body histone demethylation and reduced *FLC* transcription. The importance of *COOLAIR* splicing in this repression mechanism was confirmed by disrupting *COOLAIR* production and mutating the *COOLAIR* proximal splice acceptor site. Our findings suggest that altered splicing of a long noncoding transcript can quantitatively modulate gene expression through cotranscriptional coupling mechanisms.

INTRODUCTION

The biological significance of non-protein-coding genomic sequences has been an issue for decades (Britten and Davidson, 1969; Mattick, 2004). This has recently been reinforced by the finding that most of the human genome is represented in primary transcripts (Djebali et al., 2012). The majority of these are long, spliced, and polyadenylated RNA Polymerase II (RNA Pol II) transcripts, and a large number are antisense transcripts to annotated genes (Derrien et al., 2012; Osato et al., 2007; Lehner et al., 2002; Lu et al., 2012; Wang et al., 2005; Yamada et al., 2003). Many of the long (>200 nt) noncoding RNAs show no evolutionary conservation, adding to the debate of whether they serve any function (Gerstein et al., 2012; Graur et al., 2013).

Several in-depth studies in yeast have shown that noncoding transcripts have the potential to regulate gene expression through transcriptional interference or recruitment of chromatin modifiers (Camblong et al., 2007; Hongay et al., 2006; Castelnuovo et al., 2013). However, roles of noncoding transcripts in higher eukaryotes are less well understood. Some have been shown to play roles in chromatin regulation (Wang and Chang, 2011), although it can be the transcriptional overlap rather than the antisense transcript itself that is important for the functional consequence (Latos et al., 2012).

We have focused on the functional consequences of antisense transcription through our study of the regulation of *Arabidopsis FLOWERING LOCUS C* (*FLC*) gene, a developmental regulator that controls the timing of the switch to reproductive development. *FLC* encodes a MADS box transcriptional regulator that represses flowering, and *FLC* expression quantitatively correlates with flowering time (Sheldon et al., 1999; Michaels and Amasino, 1999). There are several regulatory pathways that converge to regulate *FLC*: two that antagonistically regulate *FLC* in ambient temperatures—the FRIGIDA pathway, which activates *FLC* expression, and the autonomous pathway, which downregulates *FLC*—and one more, vernalization, which epigenetically silences *FLC* in response to prolonged cold (Figure 1A). All of these pathways involve a set of antisense transcripts, collectively named as *COOLAIR*, that fully encompass the *FLC* gene, initiating immediately downstream of the sense strand polyadenylation site and terminating beyond the sense transcription start site (Hornyik et al., 2010; Liu et al., 2010; Swiezewski et al., 2009). *COOLAIR* transcripts are polyadenylated at multiple sites with proximal polyadenylation promoted by components of the autonomous promotion pathway. These include the RNA-binding proteins FCA and FPA, the 3′ processing factors Cstf64, Cstf77 and FY, the CPSF component and homolog of yeast Pfs2p and mammalian WDR33 (Liu et al., 2010; Ohnacker et al., 2000; Simpson et al., 2003). Use of the proximal poly(A) site results in quantitative downregulation of *FLC* expression in a process requiring FLD, an H3K4me2 demethylase (Liu et al., 2010). FLD activity results in H3K4me2 demethylation in the gene body of *FLC* and transcriptional downregulation of *FLC* (Liu et al., 2007, 2010). Loss of any of the autonomous pathway components reduces usage of the proximal polyadenylation site, which leads to increased *FLC* transcription. Analysis of the regulation of *COOLAIR* transcription has recently identified an RNA-DNA heteroduplex, or R-loop, covering the *COOLAIR* promoter (Sun et al., 2013). Stabilization of this R-loop by a novel homeodomain protein limits

COOLAIR transcription, adding another layer of regulation within the autonomous pathway.

We have continued to investigate the transcriptional circuitry at *FLC* and how *COOLAIR* is linked to changes in *FLC* expression. Here, through identification of a hypomorphic mutation in the core spliceosome component PRP8, we reveal how *COOLAIR* functionally modulates *FLC* gene expression through a cotranscriptional coupling mechanism. The *prp8* mutation reduces splicing efficiency of *COOLAIR* introns and usage of the proximal poly(A) site, increasing histone methylation in the gene body and upregulating *FLC* transcription. We also show a positive feedback mechanism between gene body histone methylation and *COOLAIR* processing. The involvement of *COOLAIR* splicing in this mechanism was supported through both disruption of *COOLAIR* production and *cis* mutation of the antisense proximal splice acceptor site. Cotranscriptional coupling mechanisms such as this may be of widespread importance in the quantitative regulation of gene expression.

RESULTS

A Hypomorphic Mutation in the Core Splicing Factor PRP8 Affects *FLC* Expression

We pursued a suppressor mutagenesis strategy to identify additional factors contributing to flowering time regulation through *FLC* repression by *FCA* (Figure 1A). We mutagenized a line that is suitable to identify factors required for FCA-mediated *FLC* repression (also referred to as C2; Liu et al., 2010). It relies on FCA overexpression (*35S-FCA*γ transgene) to enhance FCA activity and establish low levels of *FLC*, a *FLC-LUCIFERASE* (*FLC-LUC*) reporter to efficiently monitor *FLC* levels, and a functional *FRIGIDA* (*FRI*) allele to amplify changes in *FLC* expression to increase sensitivity of detection (Johanson et al., 2000). Interestingly, the commonly used *Arabidopsis* accessions such as Landsberg *erecta* (L*er*) and Columbia (Col) contain loss-of-function *fri* alleles, and the functional *FRI* we added originated from a Swedish accession (Johanson et al., 2000).

We screened for mutants with increased luciferase activity of *FLC-LUC* and identified *suppressor of overexpressed FCA* (*sof*) *81* (Figure 1B). *sof81* was a weaker suppressor than *fld*, the first mutant identified as a *sof* (Liu et al., 2007), and was found to be recessive in crosses to the C2 progenitor (Figure 1B). The mutation was mapped to *At1g80070* (Figures S1A and S1B available online), a gene that has previously been identified as essential for plant development, as null mutations lead to embryonic lethality and abnormal suspensor development (*sus* phenotype) (Schwartz et al., 1994). *At1g80070* encodes PRP8, the conserved and central component of the spliceosome (Grainger and Beggs, 2005). The *sof81* mutation changes a glycine to glutamic acid at amino acid position 1,891 (Figure 1C) within the RNase H domain of PRP8 (Figure 1D). The mutation did not change PRP8 protein levels in the plant (Figure S1C). The RNase H domain of PRP8 is thought to be an integral part of the spliceosome (Pena et al., 2008; Galej et al., 2013) that prevents premature U4/U6 unwinding and acts as a platform for exchange of U6 snRNA for U1 at the 5′ splice site (Mozaffari-Jovin et al., 2012).

The five available null alleles of *PRP8* (*sus2*) plants are embryonic lethal, indicating the mutation in *sof81* (referred to from now

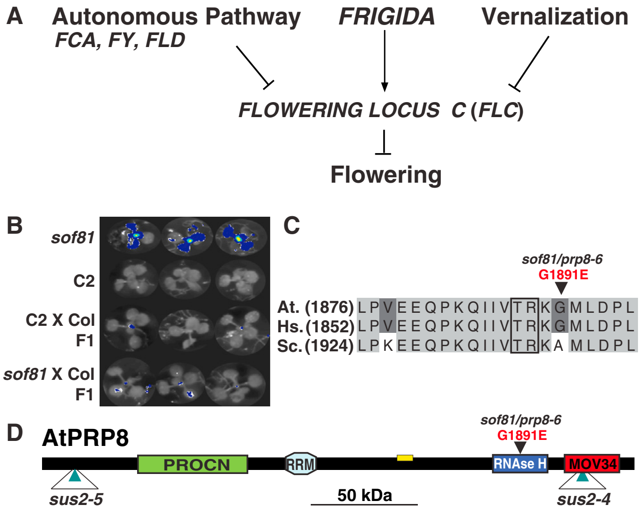

Figure 1. *sof81* Is a Mutation in the Splicing Factor PRP8 that Disrupts *FLC* Expression

(A) The different pathways that antagonistically regulate *FLC* expression are shown.

(B) Mutant screening. Three representative *Arabidopsis* seedlings of *sof81*, the C2 progenitor, and F1 individuals from *sof81* or C2 crossed to Columbia are shown. False-colored FLC-LUCIFERASE bioluminescence activity is superimposed onto the seedlings.

(C) Sequence alignment of the conserved PRP8 RNase H domain region containing the G1891E mutation in *prp8-6*. *At*, *Arabidopsis thaliana*; *Hs*, *Homo sapiens*; *Sc*, *Saccharomyces cerevisiae*.

(D) A schematic representation of the PRP8 protein. Domains and positions of different mutations are shown. Yellow box indicates the epitope of hPRP8 antibody BMR 00434 (Figure S1C). See also Figures S1 and S2.

as *prp8-6*) is hypomorphic. The *prp8-6* mutant phenotype was rescued by a genomic *PRP8* clone (Figure S2A), and heteroallelic combinations between one copy of a *prp8-sus2* allele (either *sus2-4* or *sus2-5*) and one copy of the *prp8-6* allele showed no complementation based on *FLC-LUC* bioluminescence and flowering-time analyses (Figures S2B and S2C). We therefore conclude that *prp8-6* is a recessive, hypomorphic mutation that increases *FLC* expression in *sof81*. Unlike yeast and human, *Arabidopsis thaliana* carries a second copy of *PRP8* (*At4g38780*) transcribed at low levels (Figure S1D) (Liu et al., 2009); however, given the mutant phenotype, this cannot completely cover the function of *At1g80070* in *FLC* regulation.

The *prp8* Mutation Also Affects Endogenous *FLC* Expression and Flowering Time

A similar forward mutagenesis screen had led to identification of *DCL4* as a regulator of *FCA* expression with reduction in *FCA* expression resulting in elevated levels of *FLC* (Liu et al., 2012). Therefore, we first tested whether there was any change in the expression or functionality of *FCA* in *prp8-6*. We found no change in expression of the transgene *35S-FCA*γ by western and northern blot analysis (Figures S3A and S3B). Additionally, the autoregulatory feedback limiting FCA levels was unaffected (Figures S3B and S3C) (Quesada et al., 2003). Previous data had shown that FCA associates with *FLC* chromatin (Liu et al., 2007). We found no reduction of FCA binding to the *FLC* locus

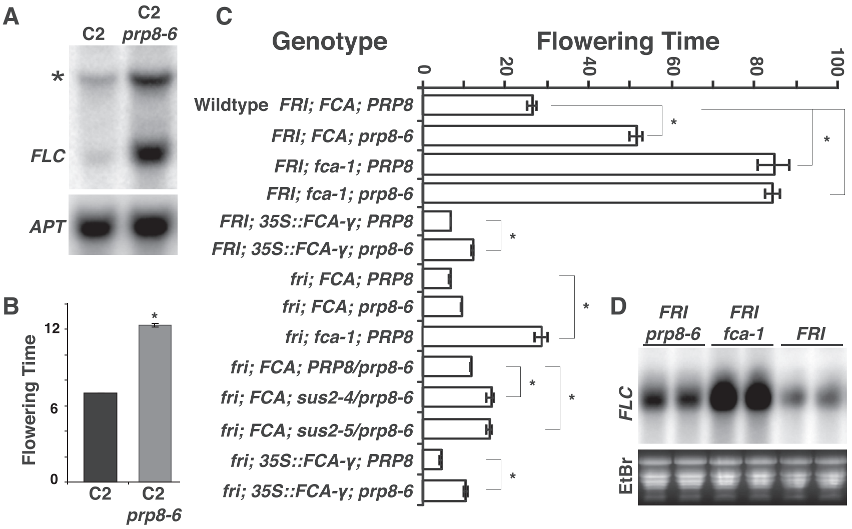

Figure 2. PRP8 Functions with FCA to Repress *FLC* and Regulate Flowering

(A) Northern analysis comparing *FLC* (*FLC*-L*er*) levels in C2 *prp8-6* to progenitor C2. The asterisk indicates the *FLC-LUC* transcript (*FLC*-Col); *APT* is the loading control.

(B) Flowering time determined by rosette leaf number at bolting, values are means ±SEM (n = 12).

(C) Flowering time phenotype of different genotypes; data determined by rosette leaf number at bolting. Values are means ±SEM (n $\geq$ 12). Student's t test was performed; p values <0.05 are denoted by (*) in (B) and (C).

(D) Northern analysis of *FLC* levels in *prp8-6* mutants and indicated controls. Two biological repeats are shown; ethidium bromide (EtBr) stained gel is loading control.

in *prp8-6*; if anything, there was an elevated level (Figure S3D). A similar lack of effect of *prp8-6* was observed on expression of other autonomous pathway components (Figure S3E); thus, we concluded that the increase of *FLC* expression by *prp8-6* is unlikely to be due to an indirect effect on autonomous pathway function.

Various polymorphisms have been reported between the *FLC* alleles of the Col and L*er* laboratory strains (*Col-FLC* and L*er*-*FLC*), including the presence of a Mutator transposon at the 3′ end of intron 1 (Liu et al., 2004). As *FLC-LUC* is based on *Col-FLC*, we tested the effect of *prp8-6* on both alleles in the same samples by northern blotting using an *FLC* probe that discriminates by size. We detected only two transcript species reflecting L*er*-*FLC* and *FLC* (*Col*)-*LUC* in *prp8-6,* both of which were increased compared to the progenitor (Figure 2A). We therefore concluded that the *prp8-6*-induced increase in expression is independent of the *cis* polymorphism between these two *FLC* alleles. We also analyzed flowering time and established that *prp8-6* delays flowering (Figure 2B), suggesting that *prp8-6* elevates biologically relevant levels of *FLC*.

We then undertook an extensive genetic study analyzing combinations of *fca-1*, *prp8-6*, and *FRIGIDA* to investigate how PRP8 influences the autonomous and FRIGIDA pathway (Figure 2C). *prp8-6* delayed the early flowering of the progenitor line (carrying the *35S-FCAγ* and *FRIGIDA* transgenes) and delayed flowering much more extensively when the *35S-FCAγ* transgene was crossed out, but was epistatic (nonadditive) with the loss-of-function mutation of *FCA*, *fca-1*. Consistent with this, when *prp8-6* was combined with just *FRI*, the expression of *FLC* was significantly higher (Figure 2D). The effect of *prp8-6* in *fri* genotypes increased when in combination with a *sus2* null allele suggesting stronger alleles than *prp8-6* would confer later flowering if they were viable (Figures 2C and S2C). The epistasis (nonadditivity) of *prp8-6* with *fca-1* indicates that *PRP8* works in the same genetic pathway as *FCA* in wild-type plants. Overall, these results suggest the *prp8-6* mutation causes a small reduction in PRP8 activity, which functions in the same genetic pathway as FCA to oppose FRIGIDA activation of *FLC*.

PRP8 Influences Sense *FLC* Expression through Effects on *COOLAIR* Splicing

PRP8 has a central role in splicing in most eukaryotes, but since the null phenotype is embryonic lethality (Schwartz et al., 1994), a role in *FLC* regulation had not previously been detected. Interestingly, mutations in two other *Arabidopsis* splicing factors, *SR45* and *PRP39-1*, have been shown to increase *FLC* expression and cause late flowering (Ali et al., 2007; Wang et al., 2007). We therefore analyzed the effect of *prp8-6* on splicing of *FLC* transcripts and also more generally. Four alternatively spliced gene models are annotated for sense *FLC* (Figure 3A). Measuring splicing efficiency of these alternative *FLC* introns by quantitative RT-PCR (qRT-PCR) indicated no effect of *prp8-6* on these splicing events (Figure 3B). No disruption of the size of the sense transcript was detected by northern blot analysis, even though a *cis* mutation in an *FLC* sense splice site in the *flc-5* mutant reveals this species (Figure S4A). The splicing efficiency of two control genes was also unaffected in *prp8-6* (*UBC9* and *EF1a*, Figure 3C).

As our analysis provided no evidence of *FLC* sense splicing defects we analyzed splicing of *COOLAIR* introns. *COOLAIR* is alternatively spliced in different environmental conditions and different genotypes (Hornyik et al., 2010; Liu et al., 2010; Swiezewski et al., 2009). We assayed the efficiency of splicing of introns present in the most abundant *COOLAIR* transcripts (schematically summarized in Figure 3A) and found it was reduced in *prp8-6* (Figures 3D). The class IIiii and IIiv forms represent <1% of the *COOLAIR* transcripts and so were not analyzed (Hornyik et al., 2010). There was no similar reduction in splicing efficiency of the class Ii intron in a mutant of another splicing regulator, *PRP39* (Figure S4B), showing that not all splicing regulators influence *FLC* expression through the same mechanism. We explored the consequences of the *prp8*-altered splicing efficiency on the poly(A) site choice in the *COOLAIR* transcript. Alternative poly(A) sites clustered in proximal and distal regions have been characterized (Hornyik et al., 2010; Liu et al., 2010; Swiezewski et al., 2009). We used a qRT-PCR assay that specifically monitored one of the proximal and one of the distal poly(A)

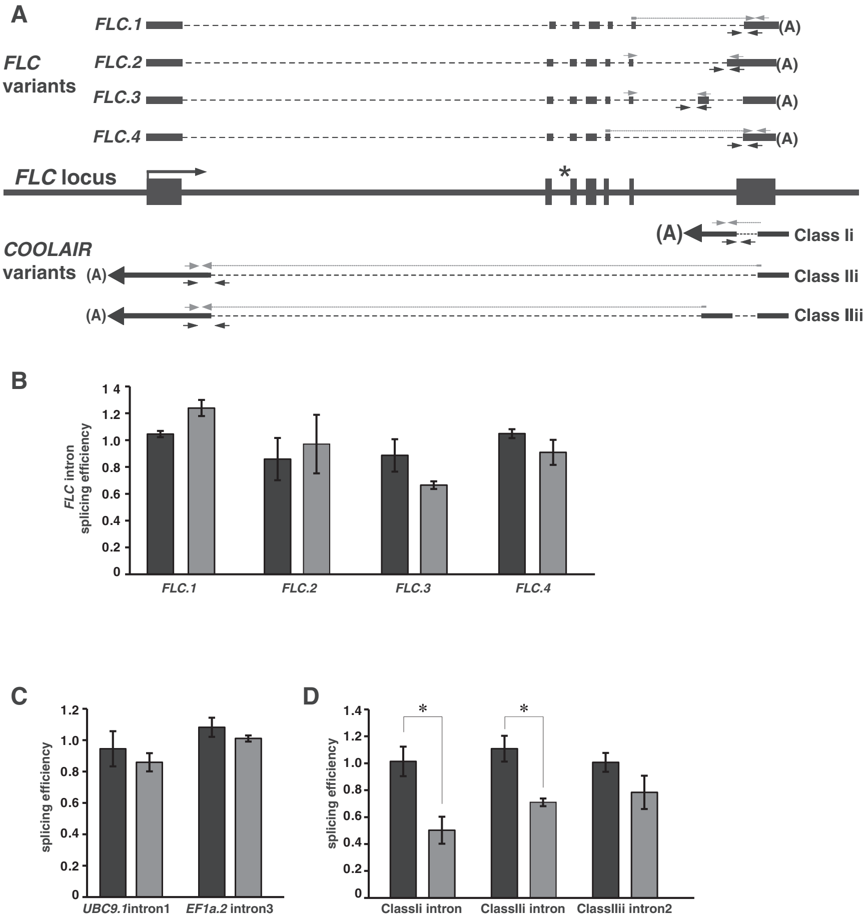

Figure 3. *prp8-6* Influences Splicing Efficiency of *COOLAIR* Introns

(A) Schematic representation of transcripts from the *FLC* locus. *FLC* and *COOLAIR* isoforms are shown including positions of primers (gray arrows and lines) used to determine splicing efficiencies. Black rectangles denote exons. The asterisk indicates the position of the *flc-5* splice acceptor *cis* element mutation of intron 2 (AG to AA) (Greb et al., 2007). Class Ii and class IIi are abundant *COOLAIR* isoforms in seedlings grown at warm temperatures, and class Ii and IIii are most abundant in cold-grown seedlings (Swiezewski et al., 2009; Liu et al., 2010; Hornyik et al., 2010).

(B) Splicing of alternative *FLC* sense introns is not affected by *prp8-6*. *FRI* in black, and *FRI prp8-6* in gray. Levels of spliced and unspliced alternative introns in *FLC* sense variants *FLC.1-4* were determined by qRT-PCR. Splicing efficiencies (spliced/unspliced) are given normalized to *FRI* background control. Details of the assays are given in extended experimental procedures in the Supplemental Information.

(C) Splicing efficiency of introns in *UBC* and *EF1a* transcripts assayed using RT-qPCR is not affected by *prp8-6* (gray), normalized to the *FRI* background control (black).

(D) Splicing efficiency of *COOLAIR* transcripts assayed using qRT-PCR of RNA from C2 (black) and C2 *prp8-6* mutants (gray).

(B–D) Values are means ±SEM (n = 3); Student's t test was performed. p values <0.05 are denoted by (*). See also Figures S3 and S4.

sites (primers shown in Table S1). *prp8-6* reduced usage of the *COOLAIR* proximal poly(A) site and promoted use of the distal site (Figure 4A). Northern blot analysis showed these data are representative of poly(A) site usage of *COOLAIR* transcripts generally (Figure S4C).

Importance of *COOLAIR* Splicing in PRP8-Dependent Repression of *FLC* Transcription

In order to explore the role of altered *COOLAIR* splicing on the *FLC* transcriptional repression mechanism, we generated an *FLC* gene where expression and splicing of *COOLAIR* was disrupted. The 3′ region of *FLC*, from the translation stop site to ~700 bp downstream, was exchanged with that from *Arabidopsis* gene *rbcs3B* (At5g38410). This generated an *FLC* transgene (named FLC^{tex}) that encoded a sense transcript with a different 3′ UTR and lacked the *COOLAIR* promoter, exon 1, and intron 1. The FLC^{tex} and wild-type *FLC* constructs were transformed into a loss-of-function *FLC* genotype (FRI *flc-2*), which has a deletion/rearrangement within the endogenous *FLC* gene (Michaels and Amasino, 1999). *FLC* transcription (assayed as spliced transcript accumulation) was ~3-fold higher in FLC^{tex} lines compared to *FLC* transgenic lines (Figure 4B). We crossed *prp8-6* into three independent, representative lines carrying the FLC^{tex} or *FLC* transgenes and assayed *FLC* expression (Figure 4B). This enabled us to compare the effect of *prp8-6* on individual transgene insertions and avoid the issue of between transgenic line expression variability. *prp8-6* did not lead to any further increases in expression in combination with FLC^{tex}. This epistasis is consistent with loss of *COOLAIR* production and *prp8-6* influencing the same mechanism.

Since reduced use of the *COOLAIR* proximal poly(A) site disrupted transcriptional repression of *FLC* (Liu et al., 2010), we reasoned that reduced splicing efficiency of the *COOLAIR* class Ii intron, necessary to generate the exon containing that poly(A) site, might be an important factor in the increased expression of *FLC* in *prp8*. We therefore specifically blocked splicing of this intron by site-directed mutagenesis of the terminal intronic dinucleotide AG to AA (Figure 5A). Mutation at this site has a minimal effect on the sense transcript, introducing one nucleotide change to the 3′ UTR downstream of the *FLC* open reading frame. Multiple, independent transgenic lines containing either wild-type *FLC* or *FLC* carrying the AG-to-AA mutation ($COOLAIR^{AA}$) were generated in a *FRI flc-2* genotype, each with and without the *prp8-6* mutation, and analyzed in pools. The AG-to-AA mutation ($COOLAIR^{AA}$) significantly reduced splicing efficiency of the intron and increased levels of *FLC* expression (Figure 5B). When combined with *prp8-6*, the AG-to-AA mutation did not further increase *FLC* levels relative to *prp8-6* alone (Figure 5B), suggesting that at least some of the *prp8-6* phenotype is the result of altered splicing of the class Ii

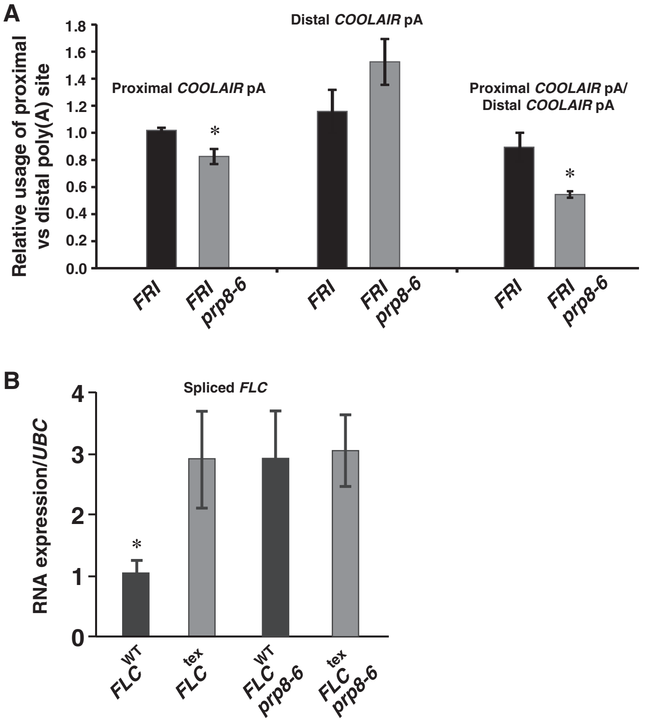

Figure 4. *COOLAIR* Plays a Role in *FLC*-Mediated Repression by PRP8

(A) Relative usage of a proximal and distal poly(A) sites of *COOLAIR* in *prp8-6*. Proximal and distal poly(A) site usage was assessed by qRT-PCR, as described in Supplemental Experimental Procedures, using primers listed in Table S1 and expressed as relative to total *COOLAIR*. Genotypes are indicated as *FRI* wild-type (black) and *FRI prp8-6* mutants (gray). Values are means from three biological repeats ±SEM.

(B) Three representative FLC^{tex} transgenic lines and two *FLC* genomic DNA transgenic controls were crossed to *prp8-6* and genotypes homozygous for *prp8-6* and all T-DNAs identified. RNA was pooled from each genotype to obtain an average expression value. Averages qRT-PCR values from three independent pooling experiments ±SEM are shown.

(A and B) Student's t test was performed. p values <0.05 are denoted by (*). See also Figure S5.

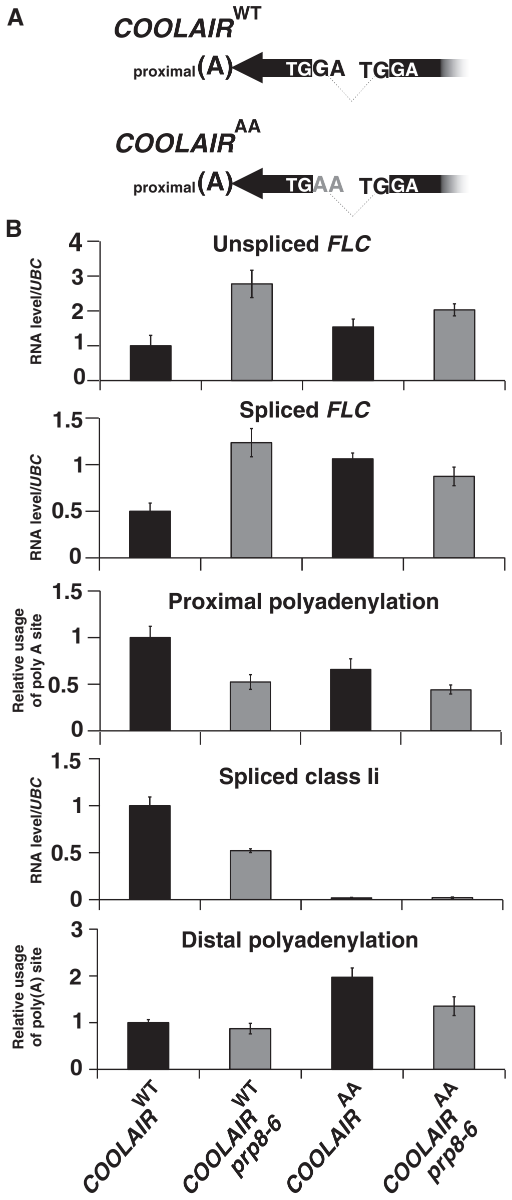

Figure 5. Mutation of *COOLAIR* Intron 1 Splice Acceptor Site Disrupts PRP8-Dependent Regulation of *FLC* Expression

(A) Schematic representation of intron 1 junction sequence in the wild-type transcript ($COOLAIR^{WT}$) or at the mutated 3′ splice site ($COOLAIR^{AA}$).

(B) qRT-PCR analysis of *FLC* unspliced and spliced RNA and relative levels of the different *COOLAIR* forms in the different transgenic genotypes. Ten to fifteen independent transgenic lines for each genotype were harvested and analyzed in pools; values are means ±SEM. qRT-PCR was used to analyze transcript levels relative to UBC. For unspliced, spliced, proximal polyadenylation, and spliced class1i, the $COOLAIR^{WT}$ value was significantly different ($p < 0.05$) from the other three genotypes. For distal polyadenylation, the $COOLAIR^{AA}$ mean is significantly higher than that of $COOLAIR^{WT}$ ($p < 0.05$). See also Figure S6.

intron. Often when the AG dinucleotide at the end of an intron is mutated downstream, AG dinucleotides are utilized instead. We used PCR with flanking primers, but we did not detect other splicing events (Figure S6A). Proximal poly(A) site usage of *COOLAIR* was reduced, and this was not additive to the *prp8-6*-induced changes (Figures 5B). Overall, these data support the view that the *prp8-6* phenotypic effects are smaller than many other autonomous pathway mutants but involve reduced splicing of *COOLAIR* class Ii intron, which reduces *COOLAIR* proximal poly(A) site usage.

Coupling of Splicing, Chromatin State, and Transcriptional Level

Alternative polyadenylation of the *COOLAIR* transcripts has been shown to trigger changes in histone methylation, increased transcription as assayed by unspliced transcript production, and RNA Pol II occupancy at the *FLC* locus (Liu et al., 2007, 2010). We therefore analyzed whether *prp8-6* influenced H3K4 demethylation and Pol II occupancy at *FLC*. *prp8-6* increased H3K4me2 in the body of the gene downstream of the proximal *COOLAIR* poly(A) site (Figures 6A and S6B), similar to changes

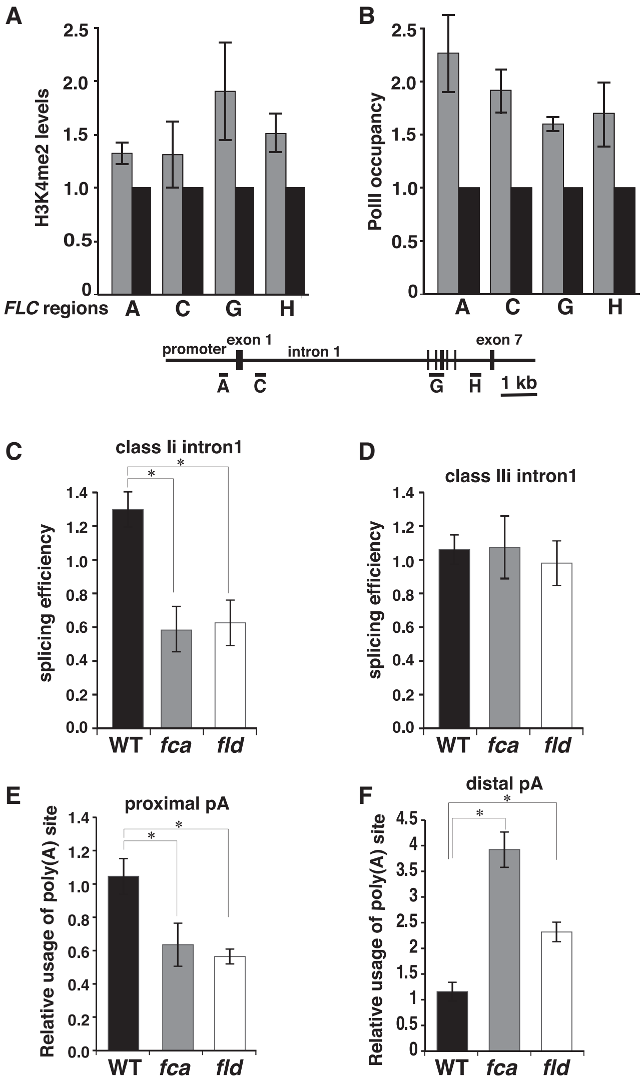

Figure 6. Coupling between PRP8 Function, Autonomous Pathway Activity, and Histone Demethylation at *FLC*

(A) Chromatin immunoprecipitation (ChIP) assays to analyze H3K4me2 enrichment at *FLC*. C2 *prp8-6* (gray) values are normalized to C2 control (black). Schematic representation of *FLC* with black rectangles denoting exons. Horizontal bars in A, C, G, and H denote regions analyzed by qPCR in ChIP experiments. H3K4me2 levels in regions A, G, and H are significantly higher in *prp8-6* than in C2 ($p < 0.05$).

(B) Pol II occupancy in different regions of *FLC* in C2 *prp8-6* (gray) normalized to the C2 control (black). PolII levels in all regions are significantly higher in *prp8-6* than in C2 ($p < 0.05$).

(A and B) Values are means ±SEM ($n \geq 3$).

(C and D) Splicing efficiency of *COOLAIR* introns is affected by *fca* (gray) and *fld* (white). Averages are from three biological repeats ±SEM in *fca-1* and *fld-6* normalized to L*er* wild-type.

(E and F) Relative usage of a proximal poly(A) site of *COOLAIR* is reduced, and a distal poly(A) site increased in *fca* and *fld* mutants. Proximal and distal poly(A) site usage is expressed as relative to total *COOLAIR*. Averages are from three biological repeats ±SEM in *fca-9* and *fld-4* normalized to Col wild-type.

(C–F) Student's t test was performed. p values <0.05 are denoted by (*). See also Figures S6 and S7.

induced by *fld* and *fca* mutations (Liu et al., 2007). We addressed whether these changes were mediated through FLD, the H3K4me2 demethylase involved in *FLC* downregulation. Consistent with a connection between PRP8 activity and FLD-induced H3K4me2 demethylation, we found that combination of the hypomorphic *prp8-6* allele with a weak *fld* mutation led to a synergistic effect on *FLC* derepression (Figure S7A). As with *fca* and *fld* mutants, the increase in H3K4me2 in *prp8-6* was associated with increased Pol II occupancy (Figures 6B, S6C, and S6D). These data support a model whereby efficient splicing of class Ii intron via PRP8 activity promotes proximal poly(A) site choice in the antisense transcript via FCA, FY activity. In turn, this proximal polyadenylation triggers FLD-mediated H3K4me2 demethylation in the gene body, which restrains transcription of *FLC*.

We then investigated how splicing and polyadenylation of *COOLAIR* might be coupled with the chromatin state at *FLC* in two ways. First, we analyzed *COOLAIR* splicing and polyadenylation in the *fld* demethylase mutant. The splicing efficiency of antisense introns class Ii was significantly reduced in *fld*, as in *fca* (Figures 6C and 6D). In addition, proximal poly(A) site usage was reduced (Figure 6E) and distal poly(A) site usage increased (Figure 6F) in an *fld* mutant. This suggested that there was positive feedback between the chromatin state at *FLC* and alternative *COOLAIR* splicing and polyadenylation. Second, we analyzed seedlings treated with the histone deacetylase inhibitor trichostatin A in order to increase the acetylation level of *FLC* chromatin. This was stimulated by the observation that *fld* mutations result in hyperacetylation of histones in *FLC* chromatin (He et al., 2003). As expected, transcriptional activity at the locus assayed by *FLC* unspliced RNA increased (Figure 7A). This was associated with an increase in total *COOLAIR* production (Figure 7B), consistent with previous data of a positive correlation between total *FLC* and total *COOLAIR* production (Swiezewski et al., 2009), and a relative reduction in proximally polyadenylated *COOLAIR* (Figure 7C). This further supported a positive feedback mechanism coupling chromatin state with *COOLAIR* processing. Chromatin modification has been proposed to affect transcript processing indirectly through influencing transcription elongation rate (Alló et al., 2009). If this is the case here, it is not dependent on the transcriptional pause release factor TFIIS (Grasser et al., 2009), because *tfIIs* mutations do not influence *COOLAIR* poly(A) site choice (Figures S7B–S7D).

DISCUSSION

The functional importance of long noncoding RNAs is a major issue in molecular biology. Analysis of the control of flowering time has enabled us to address this issue by investigating the roles of a set of long noncoding transcripts, collectively called *COOLAIR,* produced at the *Arabidopsis* locus *FLC*. *FLC* encodes a repressor of flowering whose expression level determines whether plants over-winter before flowering. Here, analysis of a hypomorphic mutation in the essential PRP8 spliceosomal subunit suggests a role for *COOLAIR* splicing in the quantitative modulation of *FLC* transcription. This hypomorphic mutation is likely to reveal the sensitivity of *FLC* regulation to changes in general function gene regulators, rather than particular specificity in PRP8 targets. Genetic and molecular analysis revealed that

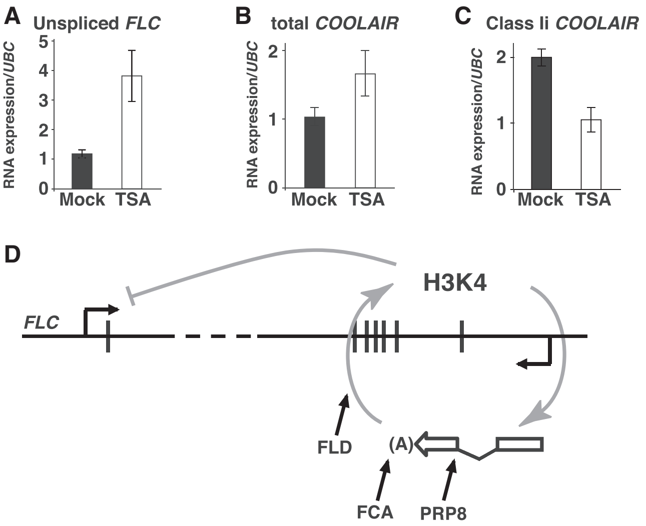

Figure 7. Cotranscriptional Coupling of Chromatin Modification with *COOLAIR* Processing

(A–C) Inhibition of histone deacetylase activity by trichostatin A (TSA) increases *FLC* expression and reduces use of the proximal *COOLAIR* polyadenylation site. *Arabidopsis* seedlings (C2 genotype) were treated (white) or not (black) with TSA. qPCR data were normalized to *UBC*. Averages are from three biological repeats ±SEM. All comparisons are statistically significant at $p < 0.05$.

(D) The autonomous pathway promotes use of the proximal antisense splice acceptor site and increases relative use of the antisense proximal poly(A) site. This results in FLD-dependent H3K4me2 demethylation in the gene body of *FLC*. We envisage that the chromatin modifications influence transcription elongation rate of both strands, leading to low *FLC* expression and reinforcing the choice of the proximal splice acceptor site of the class Ii intron and proximal polyadenylation of *COOLAIR*.

PRP8 functions in the autonomous pathway. This pathway represses *FLC* expression via promotion of *COOLAIR* proximal polyadenylation associated with gene body histone methylation changes and lower transcription. The hypomorphic mutation in PRP8 reduces the splicing efficiency of *COOLAIR* introns, reducing proximal polyadenylation and autonomous pathway function. The similar molecular phenotypes of the components of the autonomous pathway with respect to splicing, polyadenylation, and chromatin modification point to a positive feedback mechanism via cotranscriptional coupling between the chromatin methylation in the gene body and processing of the *COOLAIR* transcript (Figure 7D).

The *prp8-6* amino acid substitution is the first instance in which development of higher organisms is influenced by changes in the RNase H domain of PRP8. The viability of the mutant plants and the lack of effect on the splicing efficiency of sense *FLC* introns or other control transcripts argues for this substitution, causing only a slight reduction in PRP8 function. The effects of FRIGIDA promoting *FLC* transcription would enhance this small impairment of the autonomous pathway repression. Interestingly, specific developmental defects have been identified previously for mutations in other regions of PRP8. Retinitis pigmentosa (RP) is a heritable human disease that describes progressive degeneration of the retina during development, leading to blindness; one of the heterogeneous causes of RP is a set of mutations all clustering to the C terminus of PRP8 (Liu and Zack 2013). While the disease mechanism of RP-associated mutations in PRP8 is not fully understood, our findings suggest that a sensitivity to PRP8 may arise through cotranscriptional feedback regulation of retina regulators, particularly those associated with alternatively spliced noncoding transcripts. Exactly how the G1891E substitution impairs splicing is unknown, as is the close connection between *COOLAIR* intron 1 splicing and choice of proximal poly(A) site. Autonomous pathway mutations may be useful in the dissection of the tight connection between poly(A) site choice and last intron acceptor site choice (Martinson, 2011). Read-through transcription occurs in the *A. thaliana* genome when autonomous pathway function is impaired (in *fcafpa* double mutants) (Sonmez et al., 2011); however, these read-through products are generally spliced, resulting in poly(A) sites remaining relatively close to 3′ acceptor sites.

An important aspect of our work here has been the elaboration of the cotranscriptional coupling between *COOLAIR* and *FLC* transcription. Analysis of *fld*, the histone K4 demethylase mutant (Liu et al., 2007), and trichostatin A treatment suggested a positive feedback mechanism coupling gene body histone methylation with *COOLAIR* splicing and polyadenylation. Chromatin modifications have previously been shown to mediate alternative splicing (Batsché et al., 2006; Saint-André et al., 2011; Kornblihtt et al., 2013), but less is known about how alternative splicing induces chromatin changes. In the case of *FLC*, alternative processing of *COOLAIR* leads to histone methylation changes in the gene body, in an as yet undefined mechanism, but this coupling provides a positive feedback loop reinforcing splicing and chromatin modification outcomes. We envisage a feedback loop functioning via a kinetic coupling mechanism as proposed by Alló et al., (2009). A low expression state promoted by the autonomous pathway would be characterized by use of the proximal *COOLAIR* splice acceptor site, increased proximal polyadenylation, and FLD-dependent H3K4me2 demethylation. This state would be maintained through positive feedback with low H3K4 methylation, reinforcing use of the proximal splice acceptor site. Reduced RNA Pol II elongation rate is a likely component of this loop, as slow transcription has been linked to proximal splice site choice and early termination (de la Mata et al., 2003; Hazelbaker et al., 2013). Feedback mechanisms may generally link transcriptional elongation and alternative splicing with changed polyadenylation. For example, in the case of IgH, increased transcriptional elongation leads to read-through at a weak splice acceptor site and results in proximal polyadenylation (Martincic et al., 2009). Lariat-derived circular intronic long noncoding RNAs (ciRNAs) have recently been isolated from the nonpolyadenylated RNA population in human cells and shown to promote Pol II transcription (Zhang et al., 2013). It will be interesting to investigate if such RNAs are important in the interplay between *COOLAIR* isoforms and Pol II transcription.

Feedback mechanisms tend to produce bistable systems, as clearly demonstrated by the phenotypic heterogeneity induced through metastable epigenetic toggles in yeast cell populations (Bumgarner et al., 2012). An interesting next question is whether *FLC* exists in alternative expression states due to changes in autonomous pathway regulation. Variation in expression of the

FLC regulators both developmentally and environmentally has previously been documented. For example, one of the components of the autonomous pathway, FCA, is itself subject to negative autoregulation via alternative polyadenylation with maximal expression in the shoot and root apical meristem not reached until 5 days after germination (Macknight et al., 1997). Temperature influences several of the autonomous and FRIGIDA pathway functions (Jung et al., 2012; Blázquez et al., 2003). All these influences could then modulate the dynamics of the feedback loop so quantitatively modulating *FLC* transcription. The cotranscriptional mechanism regulating expression of the floral repressor gene *FLC* is revealing concepts of general importance to gene regulation.

EXPERIMENTAL PROCEDURES

Trans-Complementation of *sof81* with the Genomic *PRP8*

The genomic region encompassing the *PRP8/SUS2* gene on *Arabidopsis* chromosome I was inserted into a TAC library cosmid clone (pJATY50P17) that was available through the John Innes Genome Centre. A 10 kb genomic *PRP8* region was amplified by PCR with the oligonucleotides PRP8-SacII-SbfI-F and PRP8-KpnI-R using pJATY50P17 as template with Phusion DNA polymerase (NEB). The PCR fragment was cloned into the binary plant transformation vector *pCambia-1300*, conferring hygromycin resistance in plants via SbfI/KpnI cloning to generate ASM4. The cloned genomic *PRP8* region in ASM4 was sequenced to verify the absence of mutations. ASM4 was transformed into *sof81* mutants by *Agrobacterium* mediated floral-dip transformation, and hygromycin-resistant T1 transformants were isolated (n > 10). The activity of the FLC-LUC reporter of the transformants was compared to untransformed *sof81* mutant controls.

Cloning of *FLC*, *COOLAIR*^{AA}, and *COOLAIR*^{TEX}

FLC was cloned as a genomic SacI fragment (~12 kb) into the *Arabidopsis* binary vector *pCambia-1300*, which confers hygromycin resistance in plants. To generate *COOLAIR*^{AA}, fragments F1 (1,325 bp) and F2 (311 bp) were amplified from *FLC* with primers for F1 (FLC3ss_F1-forward and FLC3ss_F1-reverse) and F2 (FLC3ss_F2-forward and FLC3ss_F2- reverse) containing a mutated sequence for the 3′ splice site of *FLC* antisense class Ii intron (AA instead of AG). PCR amplification was performed with Phusion polymerase (NEB). Resulting fragments F1 and F2 with overlapping ends were fused together in 1:1 molar ratio by PCR amplification with Phusion polymerase (NEB) employing the forward primer for F1 and the reverse primer for F2. The resulting fragment was digested with NheI and BglII, gel purified, and subsequently cloned into an SphI fragment of *FLC*, replacing the wild-type NheI-BglII fragment. The resulting SphI fragment with the mutated class Ii antisense 3′ splice site was inserted into *FLC-pCambia-1300*. This mutation creates a recognition site for DraI (TTTAAA), which has been used for genotyping the hygromycin resistant transformants to verify presence of the *COOLAIR*^{AA} mutation.

F2 homozygotes of the following genotypes: *prp8-6/flc-2/FRI* and *PRP8/flc-2/FRI* were obtained from crosses of *prp8-6* and *flc-2/FRI*. The F2 homozygotes were transformed using *Agrobacterium*-mediated transformation of floral buds with the either *FLC-pCambia-1300* or *COOLAIR*^{AA}*-pCambia-1300*. The seeds from a total of 49 T1 (first generation) transformants (13 plants of *COOLAIR*^{AA}*/PRP8/flc-2/FRI*, 11 plants of *COOLAIR*^{AA}*/prp8-6/flc-2/FRI*, 15 plants of *FLC/PRP8/flc-2/FRI* and 10 plants of *FLC/prp8-6/flc-2/FRI*) were sown on GM medium without glucose and selected for hygromycin resistance (T2 generation). RNA for analysis was extracted from 4-week old seedlings.

For cloning *COOLAIR*^{TEX}, the sequence TAGCCACC that contains *FLC* translational stop TAG codon was mutagenized to create EheI restriction site TGGCGCCC. A SspI-SspI fragment containing the strong *RBCS* terminator (706 bp) was PCR amplified and cloned in sense direction between EheI and SwaI restriction sites (SwaI is located 741 bp downstream of the *FLC* stop codon, therefore replacing the corresponding genomic sequence of 3′ UTR of *FLC* and flanking downstream region to create *COOLAIR*^{TEX}).

To analyze the effect of *FLC*^{tex} seeds were collected from four homozygous plants of *FLC*^{tex}*/flc-2/prp8-6/FRI* and five homozygous plants of *FLC*^{tex}*/flc-2/PRP8/FRI*. These plants were obtained from the three independent crosses of *FLC*^{tex}*/flc-2/FRI to prp8-6*/L*er*. As a control for the *FLC*^{tex} analysis, the *flc-2/FRI* plants were transformed with *pSLJ-FLC*15 (10 kg clone of Columbia *FLC* gene) and crossed with *prp8-6*/L*er* (two independent crosses). Three plants from either *FLC/flc-2/prp8-6/FRI* or *FLC/flc-2/PRP8/FRI* were obtained. The seedlings from *FLC*^{tex} and corresponding *pSLJ-FLC* transgenic plants were grown on GM medium without glucose and BASTA resistant transformants were isolated for analysis.

Measuring *FLC* Sense Transcript

For the sense *FLC* mRNA analysis, reverse transcription was performed using *FLC* specific reverse primers with SuperScript®III Reverse Transcriptase (Invitrogen). qPCR analysis was performed on LightCycler480®II (ROCHE) with primers FLC Unspliced_LP and FLC Unspliced_RP for the unspliced sense *FLC* transcript and with primers FLC Spliced_LP and FLC Spliced_RP for the spliced sense *FLC* transcript. qPCR data was normalized to *UBC* (which was amplified with primers UBC-F and UBC-R). The primers are described in Table S1.

Measuring *COOLAIR* Splicing Efficiency

To measure the splicing efficiency of class Ii intron, 5 μg of total RNA isolated from seedlings were reverse-transcribed into cDNA, primed by Int1_RT, which is located in the exon 2 of class I and class II ii, (for locations of the primers, see also the illustration presented in Figure 3A). Resulting cDNA was used as template in qPCR reactions to amplify cDNA with the first small intron spliced by primers Int1_spliced_LP and Int1_spliced_RP, which covers the splicing junction. cDNA with the first small unspliced intron was amplified by primers Int1_unspliced_LP and Int1_unspliced_RP, which is located in the first small intron. Triplicates of all PCR reactions were performed and quantified against standard curves of cDNA dilutions. These data were then used to calculate the mean together with the spliced/unspliced ratio. RT− controls were always included to confirm absence of genomic DNA contamination.

To measure *COOLAIR* class II intron splicing efficiency, 5 μg of total RNA isolated from seedlings was reverse-transcribed into cDNA, primed by Class II unspliced F, and located in the last exon of all the class II antisense RNA. The resulting cDNA was used as template in qPCR to amplify spliced class II i with primers Class II-1_LP and Class II-1_RP, which cover the splicing junction; Class II ii intron 2 spliced with primers Class II-2_LP and Class II-2_RP, which cover the splicing junction; and *FLC* antisense big introns unspliced with primers Class II unspliced F and Class II unspliced R. Triplicate PCR reactions were performed and quantified against standard curves of cDNA dilutions before calculating the mean and spliced/unspliced ratio. RT− controls were always included to confirm absence of genomic DNA contamination.

Measuring Polyadenylated *COOLAIR*

The following primers were employed for the analysis of the *COOLAIR* transcripts: (a) for proximal poly(A) site transcript oligo(dT) primer was used for the reverse transcription and forward primer, set1_RP, and reverse primer, LP_FLCin6polyA, used for the qPCR analysis (Liu et al., 2010), and (b) for the distal poly(A) site, oligo(dT) primer was used for the reverse transcription and forward primer Set4_RP and reverse primer Set4_LP used for the qPCR analysis. qPCR reactions were performed in triplicates for each sample. Average values of the triplicates were normalized to the expression of total COOLAIR (which was amplified with Total *COOLAIR*_LP and Total COOLAIR_RP primers). The primers are summarized in the Table S1.

SUPPLEMENTAL INFORMATION

Supplemental Information includes seven figures, one table, and Supplemental Experimental Procedures and can be found with this article online at http://dx.doi.org/10.1016/j.molcel.2014.03.026.

ACKNOWLEDGMENTS

We thank all members of the Dean group for discussions. This research was supported by a EU Marie Curie PhD studentship to S.M. (MEST-CT-2005-019727), a Marie Curie Fellowship to O.R., and European Research Council Advanced Investigator (233039 ENVGENE) and BBSRC BB/D010799/1 grants to C.D.

Received: September 30, 2013
Revised: December 22, 2013
Accepted: March 4, 2014
Published: April 10, 2014

REFERENCES

Ali, G.S., Palusa, S.G., Golovkin, M., Prasad, J., Manley, J.L., and Reddy, A.S. (2007). Regulation of plant developmental processes by a novel splicing factor. PLoS ONE *2*, e471.

Alló, M., Buggiano, V., Fededa, J.P., Petrillo, E., Schor, I., de la Mata, M., Agirre, E., Plass, M., Eyras, E., Elela, S.A., et al. (2009). Control of alternative splicing through siRNA-mediated transcriptional gene silencing. Nat. Struct. Mol. Biol. *16*, 717–724.

Batsché, E., Yaniv, M., and Muchardt, C. (2006). The human SWI/SNF subunit Brm is a regulator of alternative splicing. Nat. Struct. Mol. Biol. *13*, 22–29.

Blázquez, M.A., Ahn, J.H., and Weigel, D. (2003). A thermosensory pathway controlling flowering time in *Arabidopsis thaliana*. Nat. Genet. *33*, 168–171.

Britten, R.J., and Davidson, E.H. (1969). Gene regulation for higher cells: a theory. Science *165*, 349–357.

Bumgarner, S.L., Neuert, G., Voight, B.F., Symbor-Nagrabska, A., Grisafi, P., van Oudenaarden, A., and Fink, G.R. (2012). Single-cell analysis reveals that noncoding RNAs contribute to clonal heterogeneity by modulating transcription factor recruitment. Mol. Cell *45*, 470–482.

Camblong, J., Iglesias, N., Fickentscher, C., Dieppois, G., and Stutz, F. (2007). Antisense RNA stabilization induces transcriptional gene silencing via histone deacetylation in *S. cerevisiae*. Cell *131*, 706–717.

Castelnuovo, M., Rahman, S., Guffanti, E., Infantino, V., Stutz, F., and Zenklusen, D. (2013). Bimodal expression of *PHO84* is modulated by early termination of antisense transcription. Nat. Struct. Mol. Biol. *20*, 851–858.

de la Mata, M., Alonso, C.R., Kadener, S., Fededa, J.P., Blaustein, M., Pelisch, F., Cramer, P., Bentley, D., and Kornblihtt, A.R. (2003). A slow RNA polymerase II affects alternative splicing in vivo. Mol. Cell *12*, 525–532.

Derrien, T., Johnson, R., Bussotti, G., Tanzer, A., Djebali, S., Tilgner, H., Guernec, G., Martin, D., Merkel, A., Knowles, D.G., et al. (2012). The GENCODE v7 catalog of human long noncoding RNAs: analysis of their gene structure, evolution, and expression. Genome Res. *22*, 1775–1789.

Djebali, S., Davis, C.A., Merkel, A., Dobin, A., Lassmann, T., Mortazavi, A., Tanzer, A., Lagarde, J., Lin, W., Schlesinger, F., et al. (2012). Landscape of transcription in human cells. Nature *489*, 101–108.

Galej, W.P., Oubridge, C., Newman, A.J., and Nagai, K. (2013). Crystal structure of Prp8 reveals active site cavity of the spliceosome. Nature *493*, 638–643.

Gerstein, M.B., Kundaje, A., Hariharan, M., Landt, S.G., Yan, K.K., Cheng, C., Mu, X.J., Khurana, E., Rozowsky, J., Alexander, R., et al. (2012). Architecture of the human regulatory network derived from ENCODE data. Nature *489*, 91–100.

Grainger, R.J., and Beggs, J.D. (2005). Prp8 protein: at the heart of the spliceosome. RNA *11*, 533–557.

Grasser, M., Kane, C.M., Merkle, T., Melzer, M., Emmersen, J., and Grasser, K.D. (2009). Transcript elongation factor TFIIS is involved in *arabidopsis* seed dormancy. J. Mol. Biol. *386*, 598–611.

Graur, D., Zheng, Y., Price, N., Azevedo, R.B., Zufall, R.A., and Elhaik, E. (2013). On the immortality of television sets: "function" in the human genome according to the evolution-free gospel of ENCODE. Genome Biol. Evol. *5*, 578–590.

Greb, T., Mylne, J.S., Crevillen, P., Geraldo, N., An, H., Gendall, A.R., and Dean, C. (2007). The PHD finger protein VRN5 functions in the epigenetic silencing of *Arabidopsis FLC*. Curr. Biol. *17*, 73–78.

Hazelbaker, D.Z., Marquardt, S., Wlotzka, W., and Buratowski, S. (2013). Kinetic competition between RNA Polymerase II and Sen1-dependent transcription termination. Mol. Cell *49*, 55–66.

He, Y., Michaels, S.D., and Amasino, R.M. (2003). Regulation of flowering time by histone acetylation in *Arabidopsis*. Science *302*, 1751–1754.

Hongay, C.F., Grisafi, P.L., Galitski, T., and Fink, G.R. (2006). Antisense transcription controls cell fate in *Saccharomyces cerevisiae*. Cell *127*, 735–745.

Hornyik, C., Terzi, L.C., and Simpson, G.G. (2010). The spen family protein FPA controls alternative cleavage and polyadenylation of RNA. Dev. Cell *18*, 203–213.

Johanson, U., West, J., Lister, C., Michaels, S., Amasino, R., and Dean, C. (2000). Molecular analysis of FRIGIDA, a major determinant of natural variation in *Arabidopsis* flowering time. Science *290*, 344–347.

Jung, J.H., Seo, P.J., Ahn, J.H., and Park, C.M. (2012). *Arabidopsis* RNA-binding protein FCA regulates microRNA172 processing in thermosensory flowering. J. Biol. Chem. *287*, 16007–16016.

Kornblihtt, A.R., Schor, I.E., Alló, M., Dujardin, G., Petrillo, E., and Muñoz, M.J. (2013). Alternative splicing: a pivotal step between eukaryotic transcription and translation. Nat. Rev. Mol. Cell Biol. *14*, 153–165.

Latos, P.A., Pauler, F.M., Koerner, M.V., Şenergin, H.B., Hudson, Q.J., Stocsits, R.R., Allhoff, W., Stricker, S.H., Klement, R.M., Warczok, K.E., et al. (2012). Airn transcriptional overlap, but not its lncRNA products, induces imprinted Igf2r silencing. Science *338*, 1469–1472.

Lehner, B., Williams, G., Campbell, R.D., and Sanderson, C.M. (2002). Antisense transcripts in the human genome. Trends Genet. *18*, 63–65.

Liu, M.M., and Zack, D.J. (2013). Alternative splicing and retinal degeneration. Clin. Genet. *84*, 142–149.

Liu, J., He, Y.H., Amasino, R., and Chen, X.M. (2004). siRNAs targeting an intronic transposon in the regulation of natural flowering behavior in *Arabidopsis*. Genes Dev. *18*, 2873–2878.

Liu, F., Quesada, V., Crevillén, P., Bäurle, I., Swiezewski, S., and Dean, C. (2007). The *Arabidopsis* RNA-binding protein FCA requires a lysine-specific demethylase 1 homolog to downregulate *FLC*. Mol. Cell *28*, 398–407.

Liu, M., Yuan, L., Liu, N.Y., Shi, D.Q., Liu, J., and Yang, W.C. (2009). GAMETOPHYTIC FACTOR 1, involved in pre-mRNA splicing, is essential for megagametogenesis and embryogenesis in *Arabidopsis*. J. Integr. Plant Biol. *51*, 261–271.

Liu, F., Marquardt, S., Lister, C., Swiezewski, S., and Dean, C. (2010). Targeted 3′ processing of antisense transcripts triggers *Arabidopsis FLC* chromatin silencing. Science *327*, 94–97.

Liu, F., Bakht, S., and Dean, C. (2012). Cotranscriptional role for *Arabidopsis* DICER-LIKE 4 in transcription termination. Science *335*, 1621–1623.

Lu, T., Zhu, C., Lu, G., Guo, Y., Zhou, Y., Zhang, Z., Zhao, Y., Li, W., Lu, Y., Tang, W., et al. (2012). Strand-specific RNA-seq reveals widespread occurrence of novel cis-natural antisense transcripts in rice. BMC Genomics *13*, 721.

Macknight, R., Bancroft, I., Page, T., Lister, C., Schmidt, R., Love, K., Westphal, L., Murphy, G., Sherson, S., Cobbett, C., and Dean, C. (1997). FCA, a gene controlling flowering time in *Arabidopsis*, encodes a protein containing RNA-binding domains. Cell *89*, 737–745.

Martincic, K., Alkan, S.A., Cheatle, A., Borghesi, L., and Milcarek, C. (2009). Transcription elongation factor ELL2 directs immunoglobulin secretion in plasma cells by stimulating altered RNA processing. Nat. Immunol. *10*, 1102–1109.

Martinson, H.G. (2011). An active role for splicing in 3′-end formation. Wiley Interdiscip. Rev. RNA *2*, 459–470.

Mattick, J.S. (2004). RNA regulation: a new genetics? Nat. Rev. Genet. *5*, 316–323.

Michaels, S.D., and Amasino, R.M. (1999). *FLOWERING LOCUS C* encodes a novel MADS domain protein that acts as a repressor of flowering. Plant Cell *11*, 949–956.

Mozaffari-Jovin, S., Santos, K.F., Hsiao, H.H., Will, C.L., Urlaub, H., Wahl, M.C., and Lührmann, R. (2012). The Prp8 RNase H-like domain inhibits Brr2-mediated U4/U6 snRNA unwinding by blocking Brr2 loading onto the U4 snRNA. Genes Dev. *26*, 2422–2434.

Ohnacker, M., Barabino, S.M., Preker, P.J., and Keller, W. (2000). The WD-repeat protein pfs2p bridges two essential factors within the yeast pre-mRNA 3′-end-processing complex. EMBO J. *19*, 37–47.

Osato, N., Suzuki, Y., Ikeo, K., and Gojobori, T. (2007). Transcriptional interferences in cis natural antisense transcripts of humans and mice. Genetics *176*, 1299–1306.

Pena, V., Rozov, A., Fabrizio, P., Lührmann, R., and Wahl, M.C. (2008). Structure and function of an RNase H domain at the heart of the spliceosome. EMBO J. *27*, 2929–2940.

Quesada, V., Macknight, R., Dean, C., and Simpson, G.G. (2003). Autoregulation of FCA pre-mRNA processing controls *Arabidopsis* flowering time. EMBO J. *22*, 3142–3152.

Saint-André, V., Batsché, E., Rachez, C., and Muchardt, C. (2011). Histone H3 lysine 9 trimethylation and HP1γ favor inclusion of alternative exons. Nat. Struct. Mol. Biol. *18*, 337–344.

Schwartz, B.W., Yeung, E.C., and Meinke, D.W. (1994). Disruption of Morphogenesis and Transformation of the Suspensor in Abnormal Suspensor Mutants of *Arabidopsis*. Development *120*, 3235–3245.

Sheldon, C.C., Burn, J.E., Perez, P.P., Metzger, J., Edwards, J.A., Peacock, W.J., and Dennis, E.S. (1999). The *FLF* MADS box gene: a repressor of flowering in *Arabidopsis* regulated by vernalization and methylation. Plant Cell *11*, 445–458.

Simpson, G.G., Dijkwel, P.P., Quesada, V., Henderson, I., and Dean, C. (2003). FY is an RNA 3′ end-processing factor that interacts with FCA to control the *Arabidopsis* floral transition. Cell *113*, 777–787.

Sonmez, C., Bäurle, I., Magusin, A., Dreos, R., Laubinger, S., Weigel, D., and Dean, C. (2011). RNA 3′ processing functions of *Arabidopsis* FCA and FPA limit intergenic transcription. Proc. Natl. Acad. Sci. USA *108*, 8508–8513.

Sun, Q., Csorba, T., Skourti-Stathaki, K., Proudfoot, N.J., and Dean, C. (2013). R-loop stabilization represses antisense transcription at the *Arabidopsis FLC* locus. Science *340*, 619–621.

Swiezewski, S., Liu, F., Magusin, A., and Dean, C. (2009). Cold-induced silencing by long antisense transcripts of an *Arabidopsis* Polycomb target. Nature *462*, 799–802.

Wang, K.C., and Chang, H.Y. (2011). Molecular mechanisms of long noncoding RNAs. Mol. Cell *43*, 904–914.

Wang, X.J., Gaasterland, T., and Chua, N.H. (2005). Genome-wide prediction and identification of cis-natural antisense transcripts in *Arabidopsis thaliana*. Genome Biol. *6*, R30.

Wang, C., Tian, Q., Hou, Z., Mucha, M., Aukerman, M., and Olsen, O.A. (2007). The *Arabidopsis thaliana* AT PRP39-1 gene, encoding a tetratricopeptide repeat protein with similarity to the yeast pre-mRNA processing protein PRP39, affects flowering time. Plant Cell Rep. *26*, 1357–1366.

Yamada, K., Lim, J., Dale, J.M., Chen, H., Shinn, P., Palm, C.J., Southwick, A.M., Wu, H.C., Kim, C., Nguyen, M., et al. (2003). Empirical analysis of transcriptional activity in the *Arabidopsis* genome. Science *302*, 842–846.

Zhang, Y., Zhang, X.-O., Chen, T., Xiang, J.-F., Yin, Q.-F., Xing, Y.-H., Zhu, S., Yang, L., and Chen, L.L. (2013). Circular intronic long noncoding RNAs. Mol. Cell *51*, 792–806.

Molecular Cell, Volume *54*

Supplemental Information

Functional Consequences of Splicing of the Antisense Transcript *COOLAIR* on *FLC* Transcription

Sebastian Marquardt, Oleg Raitskin, Zhe Wu, Fuquan Liu, Qianwen Sun,
and Caroline Dean

Supplemental figures legends

Figure S1. The *sof81* mutant maps to the bottom of chromosome 1 and carries a mutation in *PRP8* (*At1g80070)* that does not destabilize protein levels, related to Figure 1. (A) Mapping of the *sof81* mutation showing number of recombinant chromosomes. “MK41/42” represents an example of a polymorphic marker, at position 29.957 kb from the top of chromosome 1. High-resolution mapping is shown below. The *sof81* mutation maps between MK61/62 and MK55/56. The star (*) at MK55/56 indicates the recombinant chromosome is a different chromosome than that identified with MK61/62. (B) A dCAPS marker verifies the mutation in *sof81*. PCR amplified and BglII digested fragments are shown. The *sof81* G to A transition leads to cleavage of the PCR product. (C) Analysis of PRP8 protein levels by western blotting using the BMR00434 antibody, two biological repeats are shown. Equal loading of total protein is visualized by Coomassie staining of the membrane. (D) Gene expression analysis of the two homologous *Arabidopsis PRP8* copies. *At1g80070/SUS2* expression throughout Arabidopsis developmental stages is shown in green, *At4g38780* expression in red. Note that *At1g80070/SUS2/SOF81* expression is around 100 times higher in most stages. The figure was generated at http://jsp.weigelworld.org/expviz/expviz.jsp (Schmid et al., 2005).

Figure S2. The *prp8-6* mutation in *sof81* causes the *FLC* expression and flowering time phenotype, related to Figure 1. (A) The *prp8-6* mutation in PRP8 is required to cause the *FLC* de-repression phenotype. Genomic *PRP8* using endogenous *PRP8* promoter and terminator was cloned and transformed into *prp8-6*. FLC-LUC bioluminescence activity was determined in the transformed plants in the T1 generation. The activity of several transformants (n=12) was quantified and compared to the activity of *prp8-6* mutants (n=5) in the same experiment. A representative image displaying FLC-LUC bioluminescence activity is shown on top, quantification data is shown below as mean +/- SEM. Student’s t-test showed P-value < 0.05. Warm colours (here red in *prp8-6*) indicate high FLC-LUC bioluminescence activity. High FLC-LUC activity is reduced if a wild-type PRP8 copy is introduced into *prp8-6* through transformation. (B) The *prp8-6* mutation in *PRP8* is sufficient to cause the *FLC* de-repression phenotype. F1 complementation tests based on FLC-LUC bioluminescence activity in *Arabidopsis* seedlings (2 weeks post germination, grey) grown on plates between *prp8-6* and the strong *sus2-4* and *sus2-5 prp8* alleles. Plants heterozygous for the *sus2* T-DNA insertion alleles were crossed to *prp8-6* or C2. The F1 involving *prp8-6* but not C2 segregated for high FLC-LUC bioluminescence activity, demonstrating that the gene disrupted by the *prp8-6* mutation is allelic to gene disrupted by the *sus2* mutations. Genotyping of the individuals (results

indicated on top) confirmed that only the heteroallelic combinations with one copy of the *sus2* allele and one copy of *prp8-6* show elevated FLC-LUC bioluminescence (high activity is shown by meristematic false colouring in warm tones). Note that individuals carrying the L*er* *PRP8* wild-type copy (of the C2 parent) have low FLC-LUC bioluminescence, similar to homozygous C2. The low activity is independent of either allele contributed by the *sus2* parent (either Col *PRP8* wild-type or the *sus2* T-DNA). (C) Complementation test based on flowering time between *prp8-6* and *sus2* alleles. Flowering time analysis of individuals as in (C) assayed by counting the number of rosette leaves before bolting given as means +/- SEM. The allelic combination of *prp8-6* and *sus2* resulted in the most severe late flowering phenotype. Flowering time data for genotypes as annotated below. For the comparisons: *PRP8*-Col/*prp8-sof81* vs *prp8-sus2-4*/*prp8-sof81*; *PRP8*-Col/*prp8-sof81* vs *prp8-sus2-5*/*prp8-sof81*; *PRP8* (L*er*) vs *prp8* (*sof81*) Student's t-test showed P-values < 0.05.

Figure S3. *prp8-6* does not disrupt FCA-feedback regulation and recruitment to *FLC*, related to Figure 3.

(A) Northern analysis of transcripts derived from the 35S-*FCAγ* transgene. Total RNA was hybridised with a probe for 35S-*FCAγ*. Two major transcript species (indicated on left, read-through transcripts (Liu et al., 2012) indicated with *) are detected in C2 and *prp8-6*, none in the non-transgenic L*er*. (B) Analysis of *FCA*-auto feedback regulation. Protein extracts were analysed by western blotting and probed with FCA specific antibodies (Macknight et al., 1997). The highly abundant protein present in C2 and *C2 prp8-6* is the protein derived from the *35S-FCA*γ transgene (Quesada et al., 2003). The less abundant species with higher molecular weight detected in L*er* represents the endogenous protein; it is 10 kD longer than the transgenic protein due to a non-canonical translational start codon (Simpson et al., 2010). In C2, expression of the endogenous *FCA* is repressed by *35S-FCA*γ. The lack of endogenous FCA protein in C2 *prp8-6* shows the mutant does not disrupt FCA-feedback regulation. (C) Analysis of *FCA*-auto feedback regulation by *FCA* transcript analysis. Total RNA was hybridized with a *FCA* 5'UTR probe that specifically detects the endogenous *FCA* transcripts. Three *FCA* transcript species (indicated on left) are detected in L*er*, but only *FCAβ* is detected in C2 and *C2 prp8-6*. This indicates that *FCA*-auto feedback regulation is not sensitive to the *prp8-6* mutation. *APT* is shown as loading control (below). (D) Schematic representation of *FLC* with black rectangles denoting exons. Horizontal bars (A, C, G and H) denote regions analyzed by qPCR in chromatin immunoprecipitation (ChIP) experiments. FCA association with different *FLC* regions; ChIP assay of *prp8-6* (grey), each region normalized to C2 and calculated as the % input normalized to binding to *Actin*. Values represent means +/- SEM (n =

3). Student's t-test was performed, P-values < 0.05 for enrichment of FCA at G in *prp8-6* relative to wild type. (E) Relative expression of *FPA*, *FLD* and *LD* in Ler and *prp8-6*. 5 µg of total RNA from seedlings were reverse transcribed using a mixture of reverse primers corresponding to *FPA* (FPAex2R), *FLD* (FLDex3R) and *LD* (LD3R). Primers across the splice junctions of the intron 1 were used to measure the expression of FPA (FPA1-2F and FPAex2R). Primers across the splice junction of the intron 2 were used to measure the expression *FLD* (FLDex2-3F and FLDex3R) and *LD* (LD3F and LD3R). The expression of *FPA*, *FLD* and *LD* was normalized to *UBC*, and error bars represent SEM (n = 3). No significant differences were found at P < 0.05.

Figure S4. *FLC* expression compared to the splicing *cis*-element mutant *flc-5* and *COOLAIR* expression by northern blotting, related to Figure 3. (A) Northern analysis of *FLC* transcripts in *prp8-6* and *flc-5*. Two biological repeats are analysed, the experiment compares sense *FLC* transcripts in the presence of *prp8-6*, a *FLC* sense splice site *cis*-element mutant (*flc-5*) and L*er* wild-type. In *flc-5* the splice acceptor AG dinucleotide of *FLC* intron 2 is changed to AA (Greb et al, 2007). *APT* is shown below as loading control. High molecular weight RNA species of low abundance were detected in *flc-5* but no additional transcripts were detected in *prp8-6* or L*er*. (B) *COOLAIR* Class Ii intron1 splicing efficiency in *prp39* was not significantly different to Col-0 at P < 0.05. The splicing efficiency was measured by qPCR as described in the material and methods. Values represent biological means +/- SEM (n = 3). (C) Northern analysis of *COOLAIR* transcripts in *FRI, FRI fca-1* and *FRI prp8-6*. 40µg total RNA was loaded to detect low abundance. The blot was probed with an antisense specific single strand probe generated using primers that will hybridize to all *COOLAIR* transcripts. The two major transcript types previously characterized – class I and class II can be seen – very low abundance plus alternative splicing and heterogeneity of the exact poly (A) site contribute to the fuzziness of the hybridizing fragments. The ratio of proximal and distal polyadenylated transcripts was calculated for the analysed genotypes and displayed below to represent the relative reduction in class I in *prp8-6.* Values represent means of four biological repeat RNA preparations +/- SEM. Student's t-test showed statistically significant differences of *FRI* vs *FRI*/*fca* and *FRI* vs *FRI*/*prp8* with P-values < 0.05.

Figure S5. Design and analysis of *FLC*^{tex}, related to Figure 4. (A) Schematic representation of the *flc-2* mutant fast-neutron-induced allele (Michaels and Amasino 2001). Red indicates the genomic sequence missing in *flc-2* allele. (B) Schematic representation of the *FLC*^{tex} transgene. The *FLC*^{tex} or wild-type *FLC* transgene were transformed into *FRI flc-2* and independent lines identified.

(C) Levels of proximally polyadenylated antisense transcript were measured by RT-qPCR in *FLC-TEX* lines (QS549, #573 and #577) and controls. gFLC-v3 is a representative transgenic line carrying a wild-type *FLC* genomic clone described in (Coustham et al., 2012), the wild-type Col-0 and the *flc-2* mutant are non-transformed reference controls. Values represent means of three biological repeats +/- SEM.

Figure S6. Analysis of cryptic splicing in *COOLAIR*^{AA} and additional representations of ChIP data, related to Figures 5 and 6. (A) Mutation of the natural 3' splice site of the Class Ii *COOLAIR* intron does not lead to use of nearby cryptic 3' splice sites. The schematic representation of the *COOLAIR*^{AA} mutation is shown with primers flanking the Class Ii intron. P1 and UBC-R were used for reverse transcription (RT) and P1 with P2 for amplification. The PCR products were analyzed by agarose gel electrophoresis. The PCR fragment from the wild-type spliced transcript is absent in *COOLAIR*^{AA}, and no novel PCR fragments are detected in *COOLAIR*^{AA}. UBC amplification is shown as loading control; no amplification was observed in the no RT control. (B-D) Additional representations of the Pol II and H3K4me2 ChIP experiments shown in Figure 6A-B. (B) Representations of H3K4me2 ChIP data. Four independent ChIP experiments comparing the C2 control (black) to *prp8-6* (grey) were performed. Data is represented as % of input chromatin normalized to H3K4me2 at the Actin gene used as internal control. Bar graph shows the mean of the four experiments at each *FLC* region in (Figure S3D), error bars represent +/- SEM. (C) Representations of RNA Pol II ChIP data. Four independent ChIP experiments comparing the C2 control (black) to *prp8-6* (grey) were performed. Data is represented as % of input chromatin normalized to Pol II binding to the *Actin* gene used as internal control. Bar graph shows the mean of the four individual experiments at each *FLC* region as in (Figure S3D), error bars represent +/- SEM. (D) Technical controls for ChIP experiments. One representative sample dataset (Pol II ChIP in C2 background of one experiment) is shown. Pol II binding is represented as % of chromatin input, values of IP against Pol II are given in black (Ab) and values of no antibody controls (always performed for each sample in each experiment) are given in red on the right side of the IP bars (no Ab). As values obtained for the no antibody are much lower than those for IP against Pol II values, they are inserted into the graph. Note how Pol II binding to the constitutively transcribed *ACTIN* gene is higher than to any *FLC* region.

Figure S7. Synergistic genetic interaction between weak *fld* and *prp8* alleles and analysis of *tfIIs* mutants, related to Figure 6. (A) The *fld-6* and *prp8-6* single mutants are compared to the *fld-6/prp8-6* double mutant and the C2 background control. The FLC-LUC fusion transcript is

larger than the endogenous *FLC* transcript and marked (*). *APT* is shown as loading control. (B-D) *Arabidopsis* TFIIS does not appear to be required for *FLC* repression and proximal polyadenylation of *COOLAIR*. *FLC* transcription was analysed in *Arabidopsis tfIIs* mutants. (B) Analysis of sense *FLC* levels by RT-qPCR normalized to the *UBC* control. (C) Analysis of total *COOLAIR* transcription levels normalized to *UBC*. (D) Determining the fraction of *COOLAIR* transcripts polyadenylated at the proximal position. RT-qPCR data are means derived from three biological repeats +/- SEM. None of the means are statistically significant at $P < 0.05$.

Table S1: Oligonucleotides used in study, related to data analysis in all figures.

Name	Sequence(5’ to 3’)
Int1_RT	CTGCTGGACAAATCTCCGACAATC
Int1_spliced_LP	GACAAATCTCCGACAATCTTCC
Int1_spliced_RP	CTCACACGAATAAGGTGGCTAAT
Int1_unspliced_LP	CGACAATCTTCCGGTGACTCT
Int1_unspliced_RP	TACAAACGCTCGCCCTTATC
class II-2_LP	CTCCTCCGGCGATAAGTA
Class II-1_LP	CTCCTCCGGCGATAAGTA
Class II-1_RP	CTCACACGAATAAGAAAAGTAAAA
Class II-2_RP	ACGATAATCATAGAAAAGTAAAAGAGC
Class II-3_RP	TTGTCCAGCAGAAAAGTAAAAGA
Class II-4_LP	CCGGCGGATCTCTTGTTGT
Class II-4_ RP	AATTCTCACACGAATAAGAAAAAAACAC
Class II-4_int2_RP	AAAAACACAAACAAACACAGAACC
Class II unspliced F	TCGCTCTTCTCGTCGTCTC
Class II unspliced R	AAAACACAAACAAACACAGAACC
FLC Unspliced_LP	CGC AATTTTCATAGCCCTTG
FLC Unspliced_RP	CTTTGTAATCAAAGGTGGAGAGC
FLC Spliced_LP	AGCCAAGAAGACCGAACTCA
FLC Spliced_RP	TTTGTCCAGCAGGTGACATC
Total COOLAIR_LP	TGTATGTGTTCTTCACTTCTGTCAA
Total COOLAIR_RP	GCCGTAGGCTTCTTCACTGT
FLC3ss_F1-forward	TTTTATTGTACATCAGATATATCCTCTTCTGTGTTG
FLC3ss_F1-reverse	GCCGATTTAAAGTGGCTAATTAAGTAGTGG
FLC3ss_F2-forward	CTCTCCCACTACTTAATTAGCCACTTTAAA
FLC3ss_F2- reverse	TGTGAAGCAAACACAAGTTTTTGACAG
set1_RP	CACACCACCAAATAACAACCA
LP_FLCin6polyA	TTTTTTTTTTTTTTTACTGCTTCCA
UBC-F	CTGCGACTCAGGGAATCTTCTAA
UBC-R	TTGTGCCATTGAATTGAACCC
UBC9.1-intron1-SF	CGTGAATTCGGAAGTCTTCAA
UBC9.1-intron1-SR	GCGCTACATGAAGTAGGAGGA
UBC9.1-intron1-UF	TTTGGATCTTCTTCCCGTCTT
UBC9.1-intron1-UR	AATCCCACGATCCAAATTCC
EF1a-intron3-SR	TCCTTCTTGTCCACGCTCTT
EF1a-intron3-SF	GATTGAGAAGGAGCCCAACC
EF1a-intron3-UF	ATGGTGACGCTGGTATGGTT
FLC.1-SF	GGCTAGCCAGATGGAGAATAA
FLC.1-SR	TCAACCGCCGATTTAAGGT
FLC.1-UF	TGGTTGTTATTTGGTGGTGTG
FLC.1-UR	GGAGAGTCACCGGAAGATTG
FLC.2-SR	TGTACGATAATCATAGGTCAAAATCA
FLC.2-SF	AAAATGCTGAAAGAAGAGAACCA
FLC.2 U F	TGGTTGTTATTTGGTGGTGTG
FLC.2 UR	TCTCCATCTCAGCTTCTGCTC
FLC.3-SF	AAATGCTGAAAGAAGAGAACCAG
FLC.3-SR	ACTTCTAGACACTTGGAGTTGGA
FLC.3-UF	TTGGTTTCCTTGAAGGTTGTG
FLC.3-UR	ACTTCTAGACACTTGGAGTTGGA
FLC.4-SF	TGTTGAGAATCTTAAAGAAAAGATGG
FLC.4-SR	GGAGAGTCACCGGAAGATTG
Class I_LP	ACTGCTTCCAAACTTAAAAGC
Set4_LP	TTTTTTTTTTTTTTTGCGGTACAC
Set4_RP	GGGGTAAACGAGAGTGATGC
LB3	TAGCATCTGAATTTCATAACCAATCTCGATACAC
PRP8_3’1	AGAGACTTGTATTGCTTGTTCTTCCA

PRP8_6600F	ACCTTAGAGTCAATCACATATATGTGA
PRP8_900R	TCCAATTGAATTGGCTCCAGA
81_BglII_cCAPS_F	ACTACTTCTGTTTCTCTAGTAACAGGCTACA
81_BglII_dCAPS_R	AGCAAGTGAACCTCAAGGGGATCCAAGATC
81 MboII dCAPS F	CAAAGCAGATTATTGTCACACGGGAAG
81 MboII dCAPS R	GGCAAGCCTGGAACGGAAGC
flc-2F	GTCAAAACTCAAGCCTCAAAACC
Flc-2R	GCGTCGTGGAAGATGTGTAACTC
FCAgaF3	TGGTCATGTGGAAGATGTCTATCTCA
FCAgaR3	ACGAGGCCCTGAACCAAGTCCA
PRP8-SacII-SbfI-F	TCCCCGCGGCCTGCAGGTTCTTCAGCCTTCAGGTG ACTGT
PRP8-KpnI-R	CCGGTACCTAGAGGAGGGATGATAAAACTGCTGT
FLCexon7.5’	GGAGAATAATCATCATGTGGGAGCA
FLC3UTR.3’	CTCACACGAATAAGGTACAAAGTTC
FCA 5lead5'	GGGCTCCTAGTCCTTTGATTTCTT
FCA 5lead3'	TTGCTAGGGCTGCTTCCACGAC
FCA.E2.F	CTTTGGTGACTCTAGTGGGGATGT
FCA FR	GGAGACCCTGTGGACTGTGAAGAG
FPA1-2F	ATACGCACGACCGGCAAAAC
FPAex2R	AAAGCTGTCTTGCGTTCTCT
FLDex2-3F	GGCACAAAATCTGGTATATT
FLDex3R	CTGATCATCTTCCGCTTCAA
LD3F	TCCATTAAAATTGGAAAGCGG
LD3R	TACTAATAGCATCTTTAATTG

Extended Experimental Procedures

Genetic materials: The parental C2 line was generated by crossing L*er* containing the *35S::FCAγ* transgene, (over-expressing *FCA*) (Macknight et al., 1997), to L*er* containing transgenic *FRI* and *FLC-LUC* (Liu et al., 2007). We ensured that the three transgenes in C2 were homozygous before using the line for mutagenesis. A line (gvF) was generated in the Col background containing the same three transgenes, at the same genomic location, by crossing and then backcrossing the C2 parental line nine times to the Col wild type (Liu et al., 2010). The three transgenes were confirmed as homozygous before using it for genetic mapping of *sof81.* The *prp8* T-DNA mutant alleles *sus2-4* (ABRC stock: CS16072) and *sus2-5* (ABRC stock: CS16073) were ordered from the *Arabidopsis* stock centre (NASC) and propagated as heterozygotes. *prp8-6* was generated by crossing *sof81* to L*er*, the resulting F2 population was screened for individuals homozygous for *prp8-6* and the absence of *FRI (JU223)* and *35S::FCAγ. FRI/prp8-6* was identified from the same F2 generation; however individuals homozygous for *prp8-6* in the presence of *FRI (JU223)* but in the absence of *35S::FCAγ* were isolated. Genotyping and segregation for early flowering individuals in the F3 populations of these individuals revealed which lines were homozygous for *FRI (JU223)*. *FRI/prp8-6/fca-1* was generated by crossing *sof81* to a *prp8-6/fca-1* double mutant isolated from a cross between *prp8-6* and *fca-1*. This enabled screening the resulting F2 generation for individuals

homozygous for *fca-1* in the presence of *FRI (JU223)* but in the absence of *35S::FCAγ* to isolate the *FRI/prp8-6/fca-1* genotype. The *sof1/sof81* double mutant was generated by crossing the *sof1* mutant (Liu et al., 2007) with *sof81* and double homozygous individuals identified by genotyping.

The flowering time mutants *fca-1*, *fca-9*, *flc-5, fld-4, fld-6, flc-2* used in this study have been previously described (Greb et al., 2007; Liu et al., 2010; Liu et al., 2007; Macknight et al., 1997; Manzano et al., 2009). *tfIIs* mutants are described in (Grasser et al., 2009). *prp39* mutants are described in (Wang et al., 2007).

Mutagenesis and screen of *sof* mutants: Chemical mutagenesis of the parental line C2 was carried out as described in (Liu et al., 2007). *sof* mutants were identified in the resulting M2 population by screening for seedlings with increased FLC-LUC bioluminescence activity compared to the parental C2 control line, using the light sensitive Photek and Nightowl CCD camera systems (Liu et al., 2010).

Flowering time analysis: plants were grown in controlled environment rooms with a photoperiod of 16 hours light and 8 hours dark. Temperature ranged between 23-25 °C during the day and 20-22 °C at night. Rosette leaf number (RLN) produced by the main apical meristem before switching the developmental program to the initiation flowering was counted to measure variation in flowering time.

FLC-LUC detection: seedlings around 10-15 days after germination on plates of GM medium or soil were sprayed with 1 mM of luciferin (Promega) substrate solution and incubated in the dark at room temperature for 20 minutes. LUC bioluminescence activity of the seedlings was assayed using either the Photek or Nightowl light sensitive CCD camera detection systems. Light emitted by the FLC-LUC reporter was captured and quantified. For the purpose of representation photon counts per pixel were false coloured and superimposed on a photograph of the analysed *Arabidopsis* seedlings. Warm colours (yellow-red) represent a high FLC-LUC bioluminescence activity and cold colours (blue) represent a low FLC-LUC bioluminescence activity. A threshold of the displayed counts was chosen so that FLC-LUC bioluminescence activity of known low *FLC* expressing genotypes, always assayed alongside as internal negative controls, was only just detectable.

Genotyping:

sus2-4 was genotyped with the T-DNA left border specific oligonocleotide LB3 and PRP8_3'1 to detect the presence of the insertion. PCR using PRP8_6600F and PRP8_3'1 tested whether the T-DNA insertion was homozygous.

sus2-5 was genotyped with the T-DNA left border specific oligonucleotide LB3 and PRP8_900R to detect the presence of the insertion. PCR using PRP8_600F and PRP8_900R tested whether the T-DNA insertion was homozygous.

prp8-6 was genotyped by dCAPS marker with the oligonucleotides 81_BglII_cCAPS_F and 81_BglII_cCAPS_R followed by digestion of the PCR products with BglII and 4% agarose gel electrophoresis. BglII cleaves the mutant sequence (S1B).

An alternative method for *sof81* genotyping was using a dCAPS marker with the forward primer sof81-MboII-dCAPS-F and reverse primer sof81-MboII-dCAPS-R. Digestion with MboII resulted in 25 bp and 92 bp fragments if *sof81* was present.

flc-2 genotyping was performed with forward primer flc2-F and reverse primer flc2-R. These primers amplify the fragment that is located within the deleted region of the *flc-2*.

35S::FCAγ was genotyped by using the oligonucleotides FCAgaF3 and FCAgaR3 Presence of *35S::FCAγ* was detected by amplification of a ~200 bp product from *FCAγ* cDNA template in addition to a ~500 bp product resulting from amplification of genomic *FCA*.

Genetic mapping of *sof81*: *sof81* was backcrossed twice to the parental line to reduce the presence of interfering secondary mutations. Backcrossed *sof81* was then crossed to a Columbia line (containing the same transgenes) and the F2 screened for FLC-LUC bioluminescence. Plants with high FLC-LUC bioluminescence activity in the resulting F2 were selected and genotyped using molecular markers polymorphic between the L*er* and Col. Candidate genes lying within the final mapping interval were amplified and sequenced in the parental line as well as in the *sof81* mutants to identify specific base changes. We identified that *sof81* carries G to A transition consistent with EMS mutagenesis at position 7585 bp in the genomic sequence of *Arabidopsis PRP8* (starting the ORF-ATG). The G to A transition in *sof81* changes the GGA coding for glycine at position G1891 in the *PRP8* ORF to GAA coding for glutamic acid instead (see also Fig. 1).

Northern blot analysis: A *FLC* cDNA fragment, including the last exon and 3’ UTR, was amplified with primers FLCexon7.5’ and FLC3UTR.3’. The purified PCR product was labelled (with α-P^{32} dCTP) by a primer extension reaction, using primer FLC3UTR.3’, to generate a single-stranded antisense probe to detect *FLC* or *FLC-LUC* on total RNA blots.

The probe to analyze *FCA* feedback regulation by northern blotting was generated by first amplifying a PCR product using FCA 5lead5' and FCA 5lead3' using genomic DNA as template. The purified PCR product was labelled (with α-P^{32} dCTP) by a primer extension reaction, using the 5lead3' primer, to generate a single-stranded antisense probe to specifically detect endogenous *FCA* sense transcript species on total RNA blots.

The *COOLAIR* northern was generated using 40μg total RNA. The blot was probed with an antisense specific single strand probe generated using primers Total COOLAIR_LP and RP to amplify the DNA and Total COOLAIR_LP to synthesize the labelled probe. This probe will hybridize to all *COOLAIR* transcripts.

The probe to analyze expression of the *35S::FCAγ* transgene by northern blotting was generated by first amplifying a PCR product using fca.e2.f and FCA FR. The purified PCR product was labelled (with α-P^{32} dCTP) by a primer extension reaction, using the FCA Fr primer, to generate a single-stranded antisense probe to specifically detect transgenic *FCAγ* sense transcript species on total RNA blots. Hybridizations were performed using Ultrahyb buffer (Ambion), following the manufacturer’s instructions. The blots were stripped and re-probed with loading controls such as *APT* (Marquardt et al., 2006).

Western blot analysis to detect FCA using the KL4 antibody was performed as described (Manzano et al., 2009; Quesada et al., 2003). Slight modifications were implemented for the detection of *Arabidopsis* PRP8 using the BMR-00434 antibody. Blocking was performed in 3% BSA 1xTBS and antibody incubation in 1.5% BSA 1xTBS.

Chromatin immunoprecipitation (ChIP) was performed as described (Liu et al., 2007) using H3K4me2 antibody (Upstate, 07-030), Pol II antibody (Abcam, ab817) and anti-FCA antibody (KL4). ChIP-qPCR primers used to assay *FLC* regions are shown in Supplemental table 1. Variation in chromatin amount was accounted for by normalizing the qPCR data of IP and no antibody controls to the signal from 10% of the input chromatin (representing it as % of input). To account for differences in IP and crosslinking efficiencies between samples, the data were normalized to enrichment of the Actin locus, co-assayed as internal control (representing data as %

input/*Actin*). Four independent ChIP experiments were performed and values for each region in each experiment are derived from at least three technical repeats. Data using the %input/Actin analysis method are supplied in Figure S6, data normalized to the control genotype is used to visualize regions with greatest relative change in *prp8-6* in Figure 6.

Measuring splicing efficiency in *UBC9, EF1a* and sense *FLC.*

We measured splicing efficiency of the alternative spliced introns of *UBC9* (*At4G27960.1* intron 1) and *EF1a* (*At5g60390.2* intron 3). 5 μg of total RNA of L*er FRI* or L*er FRI prp8-6* were reverse transcribed with primers UBC9.1-intron1-SR and UBC9.1-intron1-UR that are located in the exon 2 of *At4G27960.1*, and EF1a-intron3-SR that is located in exon 4 of *At5g60390.2*. The resulting cDNA was used as template for quantitative RT-PCR reactions to measure the spliced and unspliced transcript levels. UBC9.1-intron1-UF and UBC9.1-intron1-UR were employed to measure the unspliced intron 1 of *At4G27960.1*. UBC9.1-intron1-SF and UBC9.1-intron1-SR were employed to measure the splicing junction of exon 1-exon 2 of *At4G27960.1*. EF1a-intron3-UF and EF1a-intron3-SR were employed to measure the unspliced intron 3 of *At5g60390.2*. EF1a-intron3-SFand EF1a-intron3-SR employed to measure the splicing junction of exon 3- exon-4 of *At5g60390.2*.

In order to measure the alternative splicing of sense *FLC*, 5 μg of total RNA were primed by using a mixture of primers that are listed below to specifically reverse-transcribe different forms of spliced and unspliced *FLC* sense. Primers used in the reverse transcription: FLC.1-SR located in the exon 7 of *FLC.1*; FLC.2-UR located in the exon 7 of *FLC.2*; FLC.3-SR located in exon 7 of *FLC.3*; FLC.4-SR located in the exon 6 of *FLC.4*. The primers used for the qPCR amplification are listed below. FLC.1-SF and FLC.1-SR were employed to measure the splicing junction of exon 6-exon 7 of *FLC.1*. FLC.2-SF and FLC.2-SR were employed to measure the splicing junction of exon 6 - exon 7 of *FLC.2*. FLC.3-SF and FLC.3-SR were employed to measure the splicing junction of exon 6 - exon 7 of *FLC.3*. FLC.4-SF and FLC.4-SR were employed to measure the splicing junction of exon 5 - exon 6 of *FLC.4*. FLC.1-UF and FLC.1-UR were employed to measure the unspliced intron 6 of FLC.1 and unspliced intron5 of FLC.4. FLC.2-UF and FLC.2-UR were employed to measure the unspliced intron 6 of FLC.2. FLC.3-UF and FLC.3-UR were employed to measure the unspliced intron 6 of FLC.3. Triplicates of all PCR reactions were performed and quantified against standard curves before calculating the mean. The spliced/unspliced ratio was then calculated and

normalized to the control genotype. Averages and standard errors of the spliced/unspliced ratios were calculated from data of three biological replicates for each.

Statistical analysis

We performed paired sample t-tests and denoted statistically significant differences with $p < 0.05$ in the figures throughout the manuscript by a (*), or noted the result of statistical testing in the figure legend.

Supplemental References

Manzano, D., Marquardt, S., Jones, A.M.E., Bäurle, I., Liu, F.and Dean, C. (2009) Altered interactions within FY/AtCPSF complexes required for Arabidopsis FCA-mediated chromatin silencing. Proc. Natl. Acad. Sci USA *106*, 8772-8777.

Marquardt, S., Boss, P.K., Hadfield, J., Dean, C. (2006) Additional targets of the Arabidopsis autonomous pathway members, FCA and FY. J Exp Bot. *57*, 3379-86.

Schmid, M., Davison, T.S., Henz, S.R., Pape, U.J., Demar, M., Vingron, M., Schölkopf, B., Weigel, D., Lohmann, J.U. (2005) A gene expression map of *Arabidopsis thaliana* development. Nat Genet. *37*, 501-506.

Supplemental figure 1

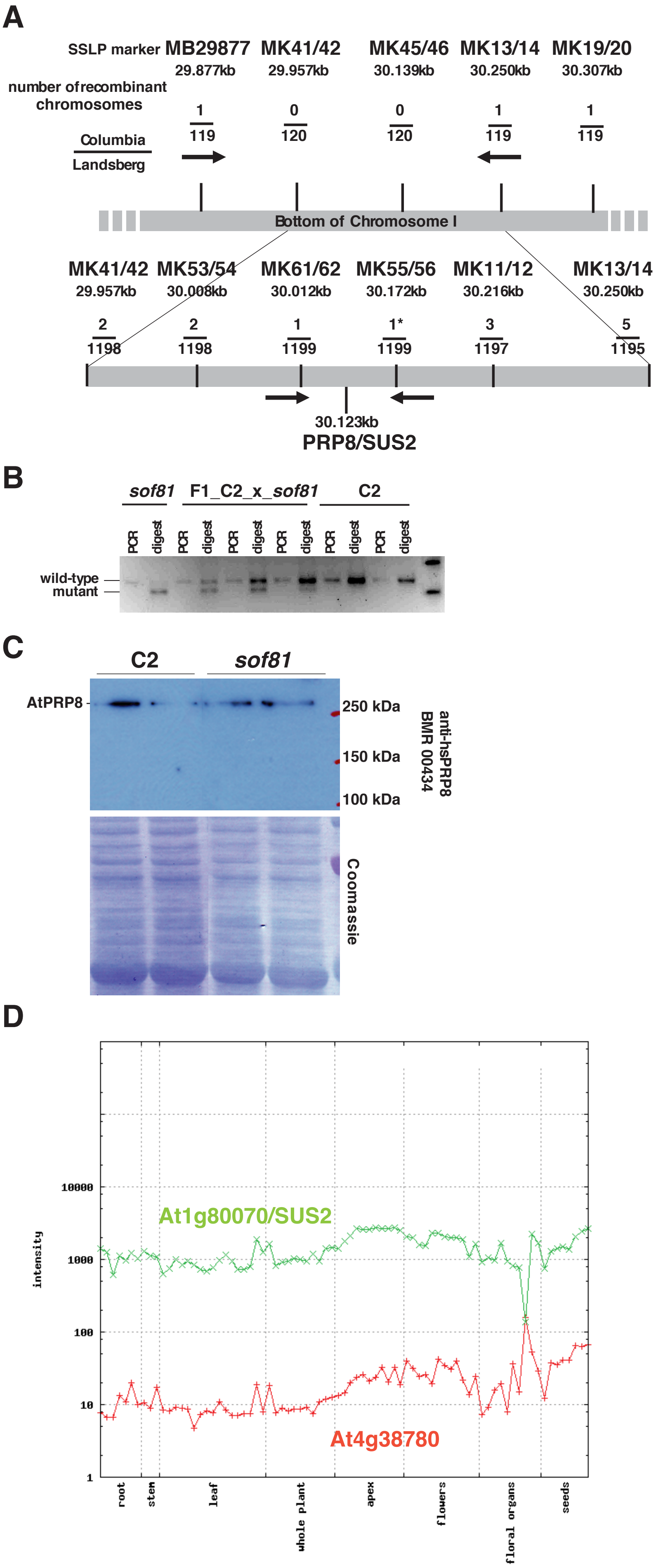

Supplemental figure 2

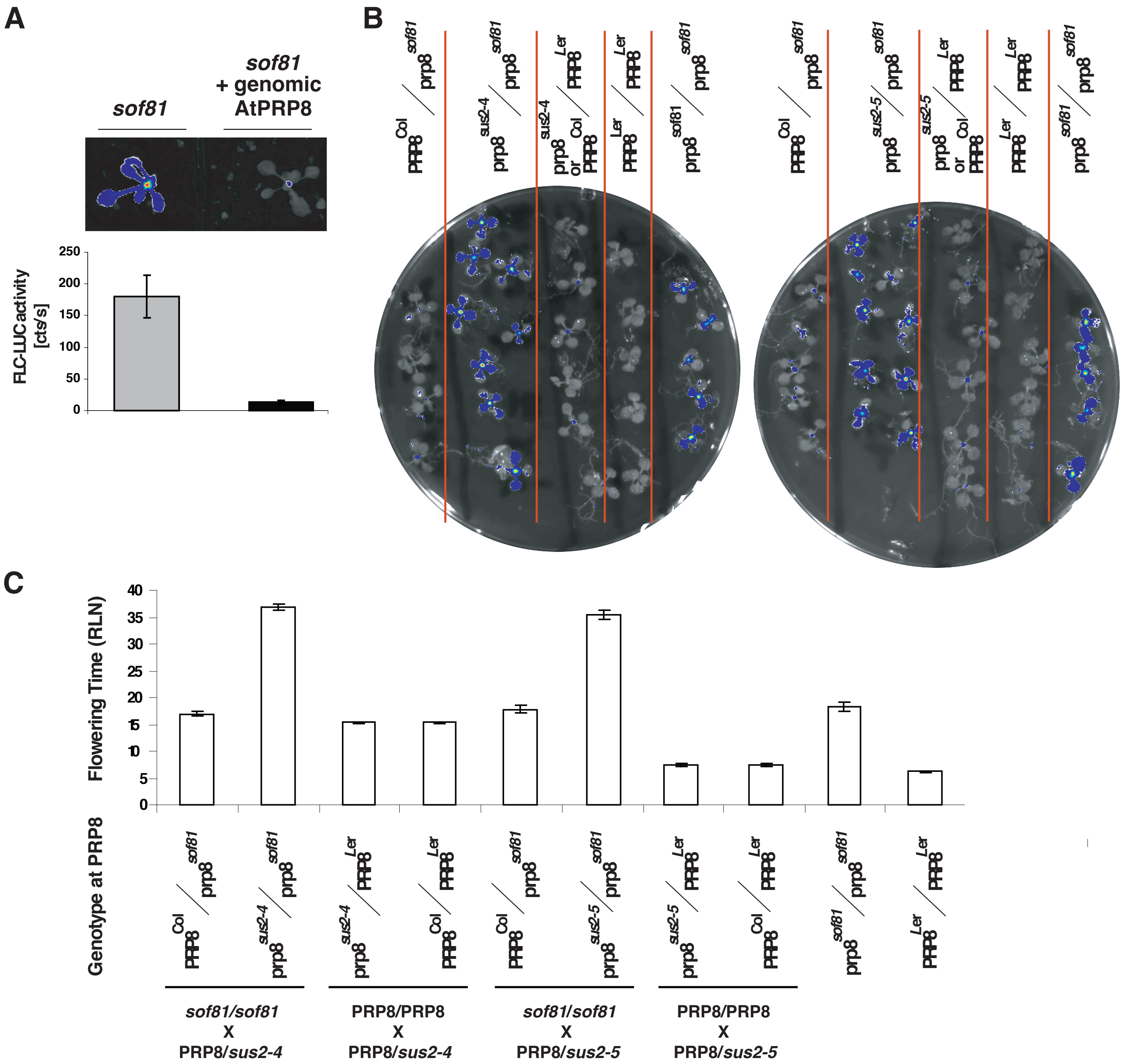

Supplemental figure 3

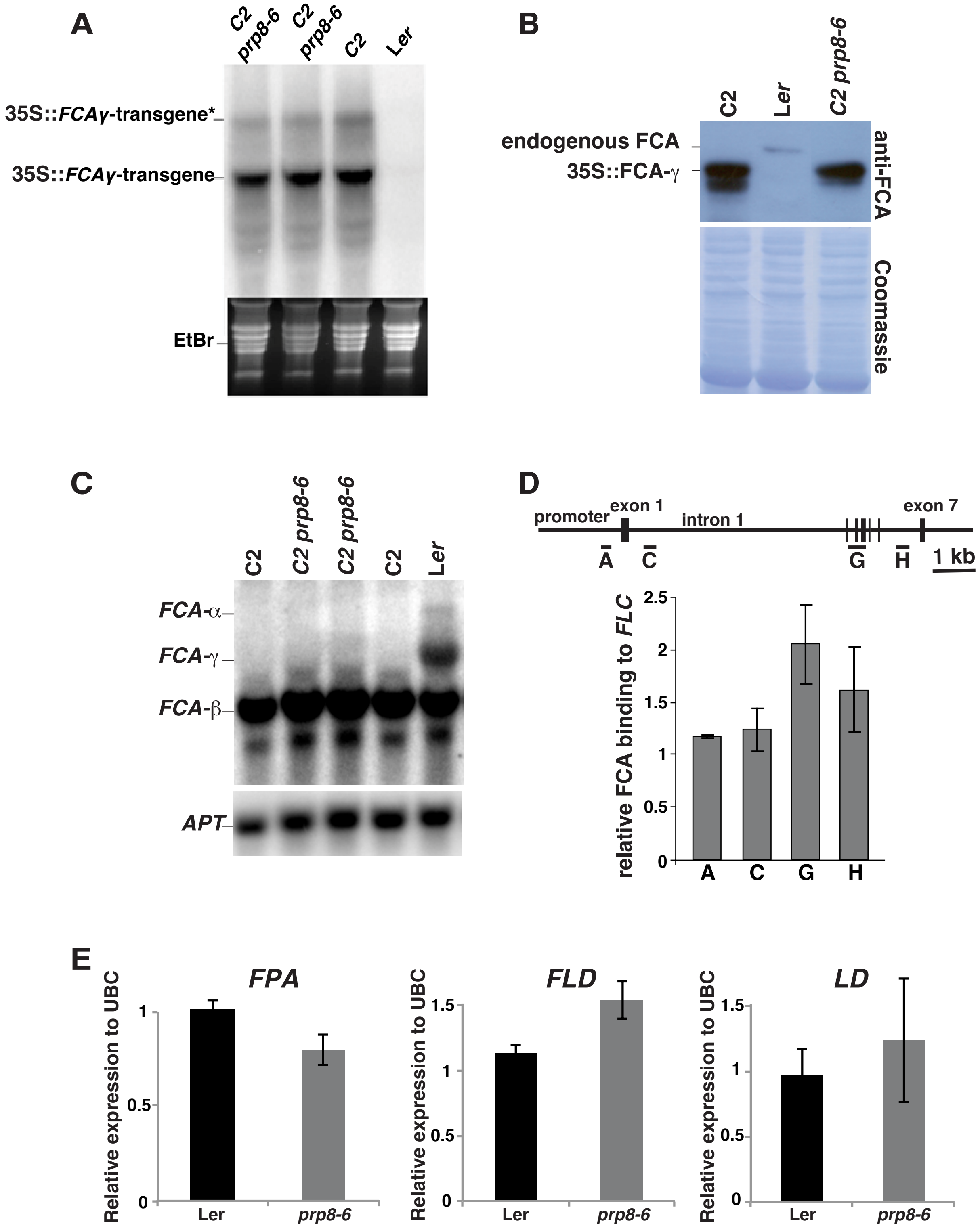

Supplemental figure 4

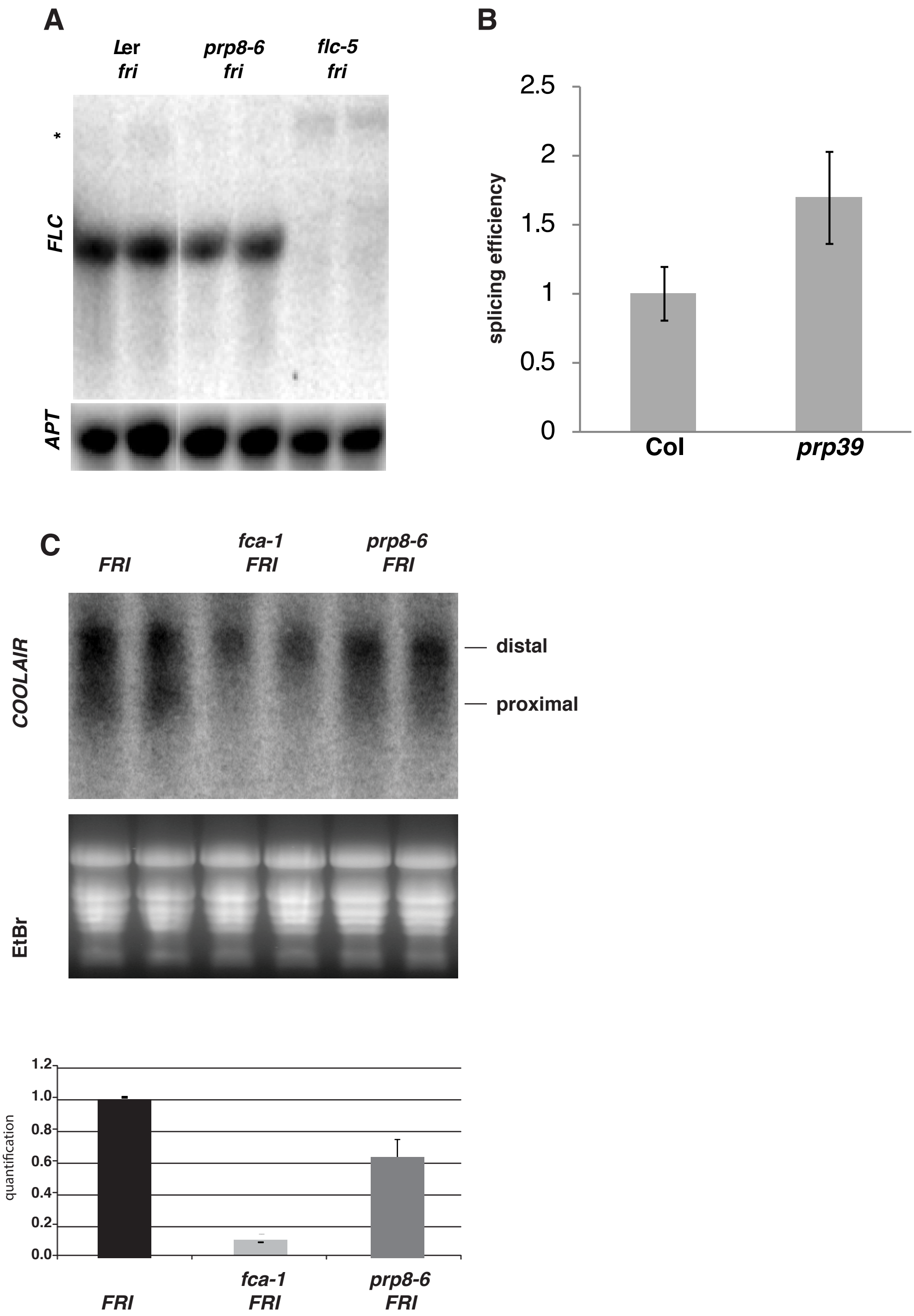

Supplemental figure 5

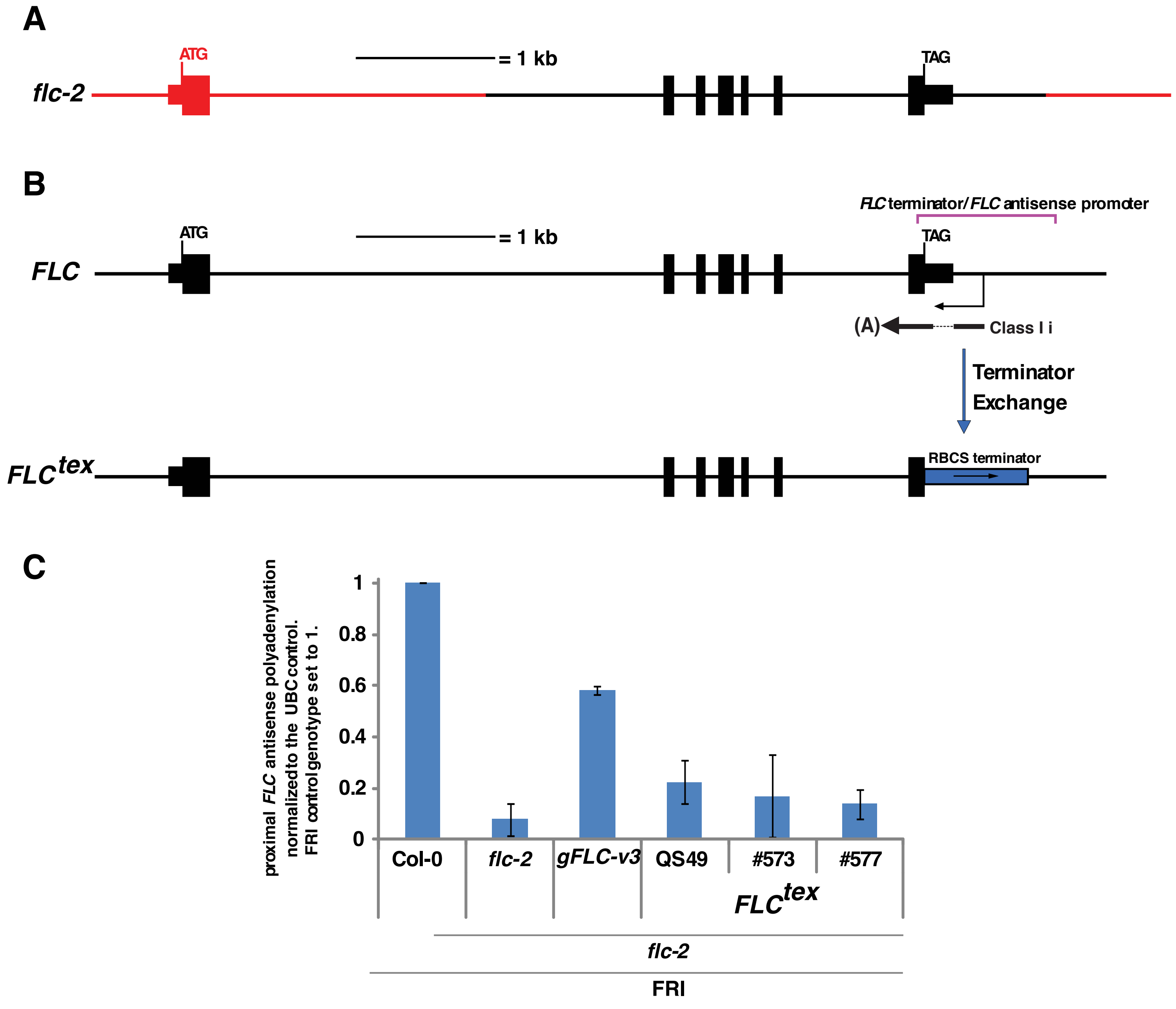

Supplemental figure 6

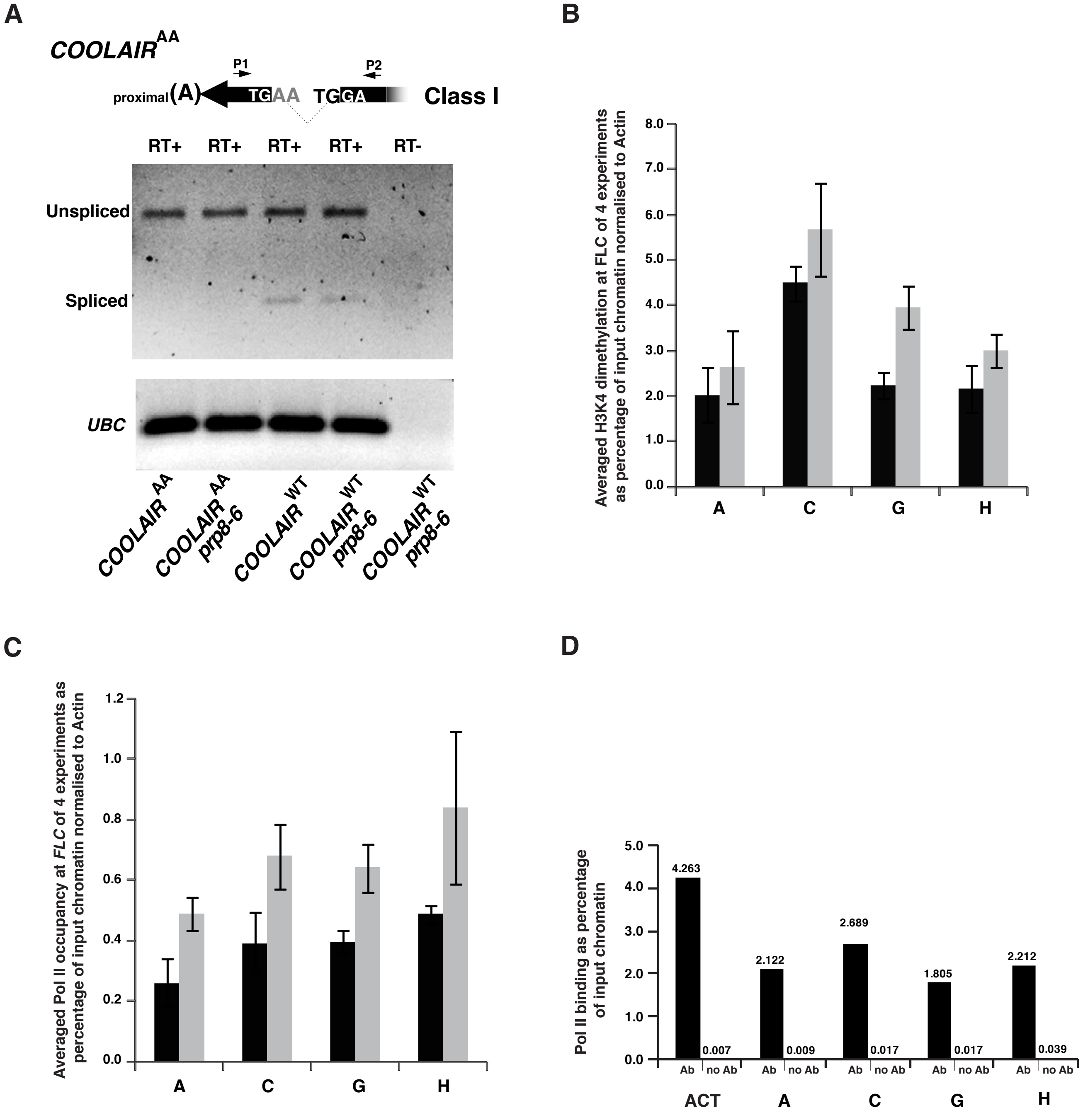

Supplemental figure 7

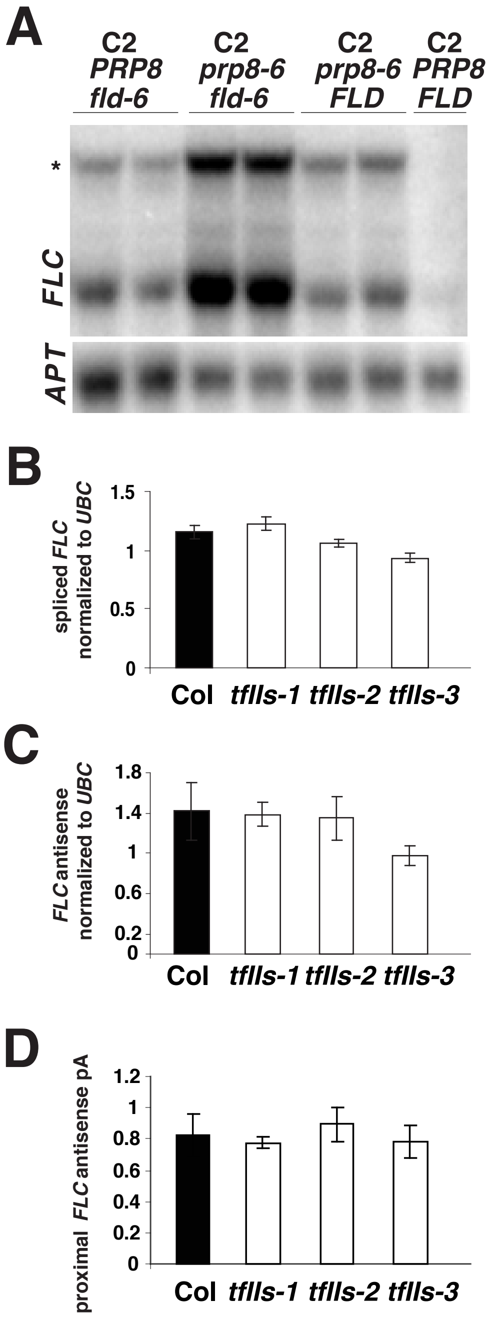

Supplement: Document S2. Article plus Supplemental Information [file mmc2.pdf]
